# Occupational exposure to silica dust and risk of lung cancer: an updated meta-analysis of epidemiological studies

**DOI:** 10.1186/s12889-016-3791-5

**Published:** 2016-11-04

**Authors:** Satiavani Poinen-Rughooputh, Mahesh Shumsher Rughooputh, Yanjun Guo, Yi Rong, Weihong Chen

**Affiliations:** 1 Department of Occupational & Environmental Health, School of Public Health, Tongji Medical College, Huazhong University of Science and Technology, Wuhan, Hubei 430030 China; 2 Key Laboratory of Environment and Health, Ministry of Education & Ministry of Environmental Protection, and State Key Laboratory of Environmental Health (Incubating), School of Public Health, Tongji Medical College, Huazhong University of Science and Technology, Wuhan, Hubei 430030 China; 3 Department of Nephrology, Tongji Hospital, Tongji Medical College, Huazhong University of Science and Technology, Wuhan, Hubei 430030 China

**Keywords:** Silica, Silicosis, Lung cancer, Meta-analysis, Heterogeneity, Meta-regression, Exposure-response analysis

## Abstract

**Background:**

Crystalline silica is considered as one of the most common and serious occupational hazards to workers’ health. Although its association with lung cancer has been studied for many decades, the conclusion remains somewhat controversial. Our objectives are to review and summarize the epidemiological evidence on the relationship between occupational silica exposure and risk of lung cancer and to provide an update on this major occupational health concern.

**Methods:**

Eligible studies up to 29 April 2016 were identified. Pooled effect estimates were calculated according to the reported outcome and the study design. Cohort, case control and proportional mortality studies were examined separately. Studies reporting results according to silicotic status were grouped together and analyzed. Due to the significant amount of heterogeneity expected, random effects models were implemented. Subgroup and meta-regression analyses (both univariate and multivariate) were performed in an attempt to explain heterogeneity. Studies which had adequate exposure characterization were selected to find out whether there was an exposure-response relationship between silica and lung cancer.

**Results:**

The risk of lung cancer was found to be elevated in both silicotics and non-silicotics. The pooled standardized mortality ratio (SMR) was 2.32 with a 95 % confidence interval (95 % CI) of 1.91–2.81 and 1.78 (95 % CI 1.07–2.96) respectively. The pooled standardized incidence ratio (SIR) was 2.49 (95 % CI 1.87–3.33) and 1.18 (95 % CI 0.86–1.62) respectively. Subgroup analysis showed that workers in the mining industry had the highest risk of lung cancer with a pooled SMR of 1.48 (95 % CI 1.18–1.86) and the weakest association was seen in potteries with a pooled SMR of 1.14 (95 % CI 1.05–1.23). A positive exposure-response relation was found between cumulative silica exposure and risk of lung cancer.

**Conclusion:**

The results of our meta-analysis supported the carcinogenic role of silica on the lungs, which was more pronounced at higher levels of exposure, in the presence of silicosis and in the mining industry. Further research is needed to evaluate whether non-silicotics are truly at risk, whether a predisposing factor would explain this potential risk, and to determine the mechanism of carcinogenicity of silica in humans.

**Electronic supplementary material:**

The online version of this article (doi:10.1186/s12889-016-3791-5) contains supplementary material, which is available to authorized users.

## Background

Crystalline silica is one of the commonest minerals on earth and a major ingredient in sand, granite, soil and glass. Traditionally, silica exposure occurs in workplaces such as coal and metal mining, metallurgy, construction industry and manufacturing of building materials, glass and clay. Recent reports indicated that more than 33 million workers in China [1] and India [2], more than 3.2 million workers in Europe [3] and about 1.7 million workers in the United States [4] are exposed to crystalline silica dust. Currently, environmental exposure to ambient silica dust caught more attention, not only during agricultural activities, but also during natural sandstorms and volcanic explosions [5, 6]. Silica exposure causes many adverse health effects including silicosis, cardiovascular diseases, tuberculosis, malignancies, autoimmune diseases and renal disorders and increased mortality, making it a high-priority public health concern [7].

The possible carcinogenicity of silica became a subject of intense debate in the scientific community in the 1980s, especially after the publication of epidemiological studies by Westerholm in 1980 [8] and Finkelstein et al. in 1982 [9], a literature review by Goldsmith et al. in 1982 [10] and presentation of new information at a 1984 symposium in North Carolina [11]. This triggered the publication of further studies on cancer mortality and morbidity in silica-exposed occupational groups. In 1997, based on a review of these studies, the International Agency for Research on Cancer (IARC) classified crystalline silica in the form of quartz or cristobalite as carcinogenic to humans (Group 1) [12]. However, the IARC working group also stated that the carcinogenicity was not found in all industrial circumstances, and their conclusion remained somewhat controversial.

The latest IARC report in 2012 reported seven meta-analyses conducted on this topic [13]. We noted that the issue of between-study heterogeneity was either not addressed at all or not dealt with in sufficient detail in these meta-analyses. Also, since the publication of the last meta-analysis on the relation between occupational silica exposure and lung cancer in 2009, more than 10 potentially relevant epidemiological studies have been conducted.

In our paper, we have combined epidemiological data from relevant studies published till date to evaluate the risk of lung cancer due to silica dust exposure and we have attempted to explain heterogeneity through subgroup and meta-regression analyses. We have also performed an exposure-response analysis by identifying studies which had well-characterized exposure data.

## Methods

The meta-analysis was conceived and performed in accordance with the Preferred Reporting Items for Systematic Reviews and Meta-Analyses (PRISMA) guidelines [14].

### Search strategy

We searched MEDLINE and EMBASE databases from January 1982 through 29 April 2016 using the search terms “lung cancer”, “silica”, “silicosis”, “risk”, “incidence” and “mortality” with variation in term construct to identify epidemiological studies published in the literature which evaluated the relationship between silica exposure and lung cancer in workers, irrespective of their silicotic status (Additional file 1). Reference lists of the identified articles were also screened for potentially eligible studies.

The following inclusion criteria were used for the analysis:

- The article had to have been published in English;
- The study had to have had a cohort or case-control or proportional mortality study design;
- Lung cancer should have been reported as a major outcome;
- The article had to have reported original results along with confidence intervals in the form of standardized mortality ratio (SMR) or standardized incidence ratio (SIR) or odds ratio (OR) or proportional mortality ratio (PMR) or mortality odds ratio (MOR) or relative risk (RR) with their corresponding 95 % confidence interval.

Reviews, autopsy studies, comments, editorials, studies with insufficient quantitative data required for the analysis (no risk estimate, no confidence intervals) and those overlapping with studies which were already considered, were excluded.

When a particular study was reported in several papers, the most recently-published reference was used unless the required data was reported in a previous paper and not in the latest-published one.

### Data extraction

For each study, the following data was extracted: geographical location, year of publication, industrial setting, study design, total number of subjects, exposure assessment (including level and duration of exposure to silica dust), outcome examined, study period (including the start date, end date and duration of follow-up), person-years of follow-up, covariates adjusted for, potential occupational carcinogens including radon, arsenic, asbestos, diesel, polycyclic aromatic hydrocarbons (PAH), talc, cadmium and amphiboles, number of lung cancer cases, total number of deaths and number of deaths due to lung cancer, measure of association and effect estimates with corresponding 95 % confidence interval (CI). Two authors worked independently for study selection and data extraction. Any disagreement was resolved after a team discussion. The list of included studies was made in consensus.

### Assessing study quality

We used the Newcastle-Ottawa Assessment Scale (NOS) for assessing the methodological quality of observational studies [15]. The scale consists of three main categories including selection of study population, comparability of subjects and ascertainment of exposure for case-control studies or ascertainment of outcome for cohort and proportional mortality studies. Scores of 0–3, 4–6 and 7–9 were assigned to low, moderate and high quality studies respectively.

### Statistical analysis

Studies were pooled together according to the outcome examined which could be incidence or mortality, the study design which could be cohort or case-control or proportional mortality study design and the measure of association. When a study reported results stratified by race, gender, industrial setting and silicotic status, they were treated as two separate reports for analysis. Studies which gave risk estimates according to silicotic status were grouped and analyzed separately. Statistical analysis was performed on the natural logarithm (ln) of the risk estimate so as to approximate its sampling distribution to a normal one. The difference between the upper and lower limits of the confidence intervals was transformed to the log scale and the standard error was calculated by dividing the transformed interval by 3.92 [16]. Random effects model was used to calculate the pooled effect estimates since a high level of heterogeneity was expected. Heterogeneity between studies was quantified by two methods namely the chi-squared test (Q test) for heterogeneity, reported by its *p* value, and the variability due to heterogeneity (I^2^ statistic), reported as a percentage in this paper [17]. We performed subgroup and meta-regression analyses to try to explain any observed between-study heterogeneity. In subgroup analysis, the studies were categorized into subgroups based on the predefined covariates. In meta-regression, we investigate the relationship between the covariates and the observed outcome [18]. The proportion of variance explained (R^2^) was used to quantify the amount of heterogeneity accounted for by each covariate. It was calculated as the percentage of ratio of variance explained to the total amount of variance. Both univariate and multivariate meta-regression models were used to try to lower the variability due to heterogeneity (I^2^) to the minimum level and to bring the *p* value of Q close to 1. Heterogeneity should be completely absent (I^2^ is 0 and *p* value of Q is 1) for an ideal comparison [19]. Differences in exposure assessment, study design and quality, data collection processes, outcome assessment, selection of subjects and definition of confounding factors often account for significant between-study heterogeneity [16]. Based on this statement, the covariates considered were year of publication, presence of at least one confounding factor, adjustment for smoking, industrial setting, geographical location, NOS score, cumulative silica dust exposure level, duration of exposure, concentration of silica dust, person-years of follow-up, number of subjects and total number of deaths. For sensitivity analyses, we assessed the influence of individual studies on the pooled estimate by omitting each study in turn (leave-one-out analysis). Publication bias was assessed graphically by means of funnel plots and quantitatively by Egger’s linear regression method [20]. For the exposure-response analyses, we used the average cumulative silica dust exposure as covariate and the risk estimate of the corresponding study as the effect. No imputation was made in relating the effect estimate to the exposure level. Statistical analysis was done using R software version 3.1.2 (2014-10-31) [21] with the ‘metafor’ package version 1.9-5 [22].

The levels of significance for all statistical tests were assumed to be equal to or less than 0.05, except in the case of heterogeneity testing whereby the level of significance was assumed to be equal to or less than 0.10 [17].

## Results

### Characteristics of studies and bias assessment

**Fig. 1:**
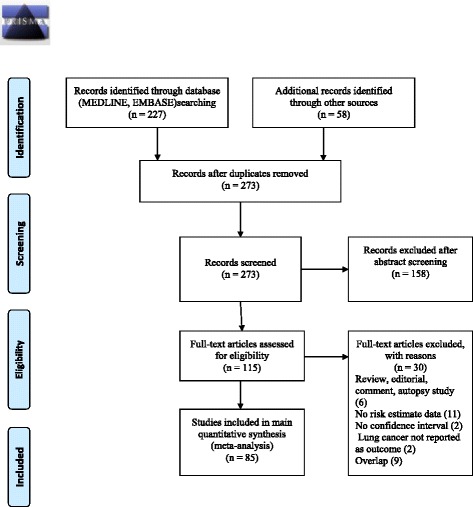
The PRISMA flowchart for the selection of studies

The PRISMA flowchart for the selection of studies is shown in Fig. 1. The initial search criteria yielded 227 citations from the databases. 58 additional records were further identified from references of related articles. After removing duplicates, we were left with 273 records. Preliminary screening of abstracts eliminated 158 studies. Of the remaining 115 articles, 30 were excluded for the following reasons: 6 articles were found to be either reviews, editorials, comments or autopsy studies, 11 papers had no risk estimate data, 2 articles did not give the confidence intervals of the effect estimate, 2 articles did not report lung cancer as outcome and 9 articles had overlapping populations with selected studies. After exclusion of these 30 studies, 85 articles were left and included in the final main quantitative synthesis [9, 10, 23–104]. The study of Puntoni et al. [105], which was excluded from the main synthesis due to overlap with the study cohort of Merlo et al. [66], was included in the list of silicotic studies since it contained the risk estimate based on silicotic status whereas the study by Merlo et al. had the risk estimate of the whole cohort and not according to silicotic status.

After categorization of the studies by outcome assessed, study type and measure of association there were 63 cohort studies reporting mortality due to lung cancer in the form of SMR as measure of association, 19 cohort studies reporting incidence of lung cancer in the form of SIR, 1 cohort study reporting incidence in the form of RR, 3 case-control studies reporting mortality in the form of MOR, 9 case-control studies reporting incidence in the form of OR, 5 case-control studies reporting mortality in the form of OR and 2 proportional mortality studies or PMR. The characteristics of all included studies are shown in Additional file 2.

Most studies comprised males only and a mere few included both males and a small proportion of females (around 10 %). Only exceptions were the studies of Zhang et al. [104] and Smailyte et al. [84] with 26 and 31 % women respectively. Nine papers reported 2 or more results stratified by industrial setting, sex, silicotic status and racial background. Forty-one studies were conducted in European countries, 18 in the United States, 21 in Asian countries, 9 in Canada, 3 in Australia and 1 in South Africa. The industries of concern were mining, foundry, pottery and ceramic, refractory brick and diatomaceous earth processing, granite which included sand and quarry, cement production and construction. The total number of studies available for analysis according to silicotic status was 34. The characteristics of silicotic and non-silicotic studies are described in Table 1.

**Table 1:** Characteristics of silicotic and non-silicotic studies

	Author, Year	Country	Industry	Covariates adjusted for	Number of subjects	Outcome examined	Measure, silicotic status	Effect Estimate (95 % CI)	Observed lung cancer deaths or cases
	Cohort studies								
1	Amandus, 1995 [25]	USA	Mixed	Age, sex, race, talc, asbestos	760	Mortality	SMR, silicotic	2.30 (1.50–3.40)	
2	Berry, 2003 [28]	Australia	Mixed	Age, sex, calendar period, smoking	1467	Mortality	SMR, silicotic	1.90 (1.50–2.30)	94
3	Carta, 2001 [31]	Sardinia	Mine & quarries	Age, sex, calendar period	724	Mortality	SMR, silicotic	1.37 (0.98–1.91)	34
4	Chan, 2000 [33]	Hong Kong	Mixed	Age, sex, calendar period	1502	Mortality	SMR, silicotic	1.94 (1.35–2.70)	33
5	Chen, 1992 [34]	China	Mixed	Age, sex	70179	Mortality	SMR, silicotic	1.22 (0.90–1.60)	
6	Chen, 1990 [35]	China	Iron mine	Age, sex	1226	Mortality	SMR, silicotic	5.30 (2.90–8.80)	14
						Mortality	SMR, non-silicotic	2.90 (1.60–4.70)	15
7	Chen, 2006 [37]	China	Mine	Age, sex	932	Mortality	SMR, silicotic	4.13 (3.15–5.29)	
						Mortality	SMR, non-silicotic	1.96 (1.50–2.73)	
8	Chia, 1991 [39]	China	granite	Age, sex, calendar period	159	Incidence	SIR, silicotic	2.01 (0.92–3.81)	9
9	Chiyotani, 1990 [40]	Japan	Mixed	Age, sex	1941	Mortality	SMR, silicotic	6.03 (5.29–6.77)	44
10	Finkelstein, 1982 [10]	Canada	mine	Age, sex, calendar period	1190	Mortality	SMR, silicotic	2.30 (1.80–3.00)	62
11	Finkelstein, 1995 [43]	Canada	Mixed	Age, sex	328	Incidence	SIR, silicotic	2.55 (1.43–8.28)	15
						Incidence	SIR, non-silicotic	0.90 (0.51–1.47)	16
12	Goldsmith, 1995 [49]	USA	Mixed	Age, sex, calendar period	590	Mortality	SMR, silicotic	1.90 (1.35–2.60)	39
13	Infante- Rivard, 1989 [54]	Canada	Mixed	Age, sex, calendar period	1072	Mortality	SMR, silicotic	3.47 (3.11–3.90)	83
14	Marinaccio, 2006 [63]	Italy	Mixed	Age, sex, calendar period	14929	Mortality	SMR, silicotic	1.10 (1.03–1.18)	798
15	Mehnert, 1990 [64]	Germany	quarry	age, sex	2475	Mortality	SMR, silicotic	1.83 (0.84–3.48)	9
						Mortality	SMR, non-silicotic	0.91 (0.54–1.44)	18
16	Merlo, 1995 [67]	Italy	Mixed	Age, sex, calendar period	450	Mortality	SMR, silicotic	3.50 (2.44–4.87)	35
17	Ng, 1990 [71]	Hong Kong	Mixed	Age, sex, PAH, asbestos	1419	Mortality	SMR, silicotic	2.03 (1.35–2.93)	28
18	Partanen, 1994 [73]	Finland	Mixed	Age, sex, calendar period	811	Incidence	SIR, silicotic	2.89 (2.35–3.48)	190
19	Puntoni, 1988 [105]	Italy	Refractory brick	Age, sex	231	Mortality	SMR, silicotic	1.67 (0.61–3.64)	6
						Mortality	SMR, non-silicotic	2.08 (0.67–4.84)	5
20	Scarselli, 2011 [81]	Italy	Mixed	Age, sex, calendar period	2034	Mortality	SMR, silicotic	1.39 (1.17–1.64)	139
21	Sherson, 1991 [83]	Denmark	Foundry	Age, sex, calendar period	6144	Incidence	SIR, silicotic	1.71 (0.85–3.06)	11
						Incidence	SIR, non-silicotic	1.30 (1.07–1.47)	150
22	Tornling, 1991 [88]	Sweden	Ceramic	Age, sex	280	Mortality	SMR, silicotic	2.36 (1.07–4.48)	9
23	Tse, 2014 [90]	Hong Kong	Mixed	Age, sex, calendar period	3202	Mortality	SMR, silicotic	1.86 (1.59–2.17)	157
24	Wang, 1996 [96]	China	Metallurgy	Age, sex, calendar period	4372	Mortality	SMR, silicotic	2.37 (1.96–2.86)	104
25	Westerholm, 1980 [9]	Sweden	Mixed	Age, sex, calendar period	3610	Mortality	SMR, silicotic	3.80 (2.30–5.80)	
26	Westerholm, 1986 [99]	Sweden	Mixed	Age, sex, calendar period	712	Mortality	SMR, silicotic	5.38 (2.20–11.10)	7
27	Yu, 2008 [102]	Hong Kong	mixed	Age, calendar period, smoking	2798	Mortality	SMR, silicotic	1.56 (0.98–2.63)	86
28	Zambon, 1987 [103]	Italy	Mixed	Age, sex, calendar period	1313	Mortality	SMR, silicotic	2.39 (1.86–3.02)	70
	Case-control studies								
1	Forastiere, 1989 [45]	Italy	Mixed	Age, sex, calendar period	595	Mortality	MOR, silicotic	2.50 (1.20–4.60)	10
2	Fu, 1994 [46]	China	Tin mine	Age, sex, smoking	267	Incidence	OR, silicotic	2.03 (1.25–3.29)	
3	Lagorio, 1990 [61]	Italy	Pottery	Age, calendar period, smoking	391	Mortality	OR, silicotic	3.90 (1.80–8.30)	
						Mortality	OR, non-silicotic	1.40 (0.70–2.80)	
4	Neuberger, 1988 [70]	Austria	Mixed	Age, sex, calendar period, area, smoking	2212	Mortality	MOR, silicotic	1.41 (1.21–1.64)	182
5	Schuller, 1986 [82]	Switzerland	Mixed	Calendar period	2399	Mortality	MOR, silicotic	2.23 (1.90–2.60)	180
6	Tsuda, 2002 [91]	Japan	Mixed	Age, sex, smoking	501	Mortality	OR, silicotic	2.77 (1.60–4.77)	184

*CI* confidence interval, *USA* United States of America, *PAH* polycyclic aromatic hydrocarbons, *SMR* standardized mortality ratio, *SIR* standardized incidence ratio *OR* odds ratio, *MOR* mortality odds ratio

The results of the study quality assessment are presented in Additional file 3. Ten articles were deemed to be of low quality, 49 articles were found to be of medium quality and 26 articles were shown to be of high quality. The median score for all 85 articles was 5.3 out of a maximum of 9.

As demonstrated graphically by the funnel plots in Additional files 4, 5 and 6, there was evidence of publication bias for studies reporting mortality in the form of SMR (*p* = 0.024 for Egger’s regression test) but no evidence of publication bias for studies reporting incidence in the form of SIR (*p* = 0.238) and OR (*p* = 0.457).

### Data analysis

**Fig. 2:**
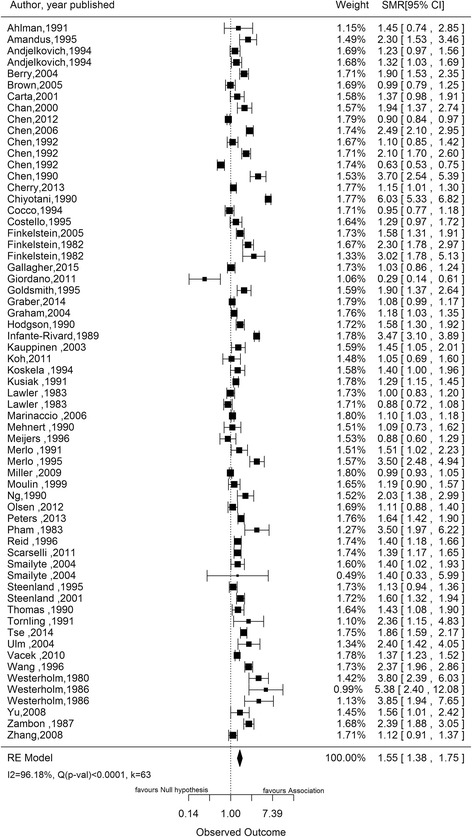
Forest plot showing pooled standardized mortality ratio (SMR) of lung cancer due to silica dust. SMR, Standardized mortality ratio; RE, Random effect; I^2^, Variability due to heterogeneity; Q, Chi-square test for heterogeneity; K, Number of studies

**Fig. 3:**
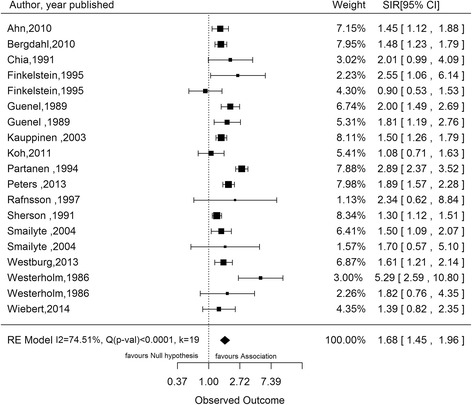
Forest plot showing pooled standardized incidence ratio (SIR) of lung cancer due to silica dust. SIR, Standardized incidence ratio; RE, Random effect; I^2^, Variability due to heterogeneity; Q, Chi-square test for heterogeneity; K, Number of studies

**Fig. 4:**
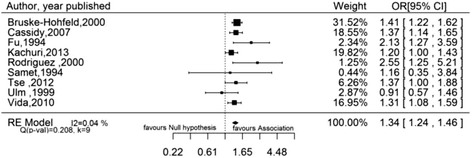
Forest plot showing pooled odds ratio (OR) of lung cancer due to silica dust. OR, Odds ratio; RE, Random effect; I^2^, Variability due to heterogeneity; Q, Chi-square test for heterogeneity; K, Number of studies

Using the random effects model, the pooled estimate was 1.55 (95 % CI 1.38–1.75) for SMR studies, 1.68 (95 % CI 1.45–1.96) for SIR studies, 1.10 (95 % CI 0.89–1.36) for PMR studies, 1.69 (95 % CI 1.26–2.26) for MOR studies, and 1.34 (95 % CI 1.24–1.46) for case-control studies reporting incidence as outcome and 1.82 (95 % CI 1.25–2.66) for case-control studies reporting mortality as outcome. The risk estimate in each category was statistically significant (*p* < 0.05) except in the category of PMR studies (*p* = 0.38). The results of the SMR, SIR and OR studies with incidence as outcome are illustrated in forest plots in Figs. 2, 3 and 4 respectively. Significant between-study heterogeneity was observed in SMR, MOR and SIR studies with I^2^ of 96 %, 87 % and 75 % respectively. PMR and OR studies with mortality as outcome showed lower between-study heterogeneity (I^2^ 62 % and 51 % respectively), which was statistically insignificant (*p* value for Q test >0.10 for both). No heterogeneity was observed in the meta-analysis of OR studies with incidence as outcome. Studies conducted in silicotic subjects yielded a significantly higher pooled SMR of 2.32 (95 % CI 1.91–2.81) and SIR of 2.49 (95 % CI 1.87–3.33) as compared to non-silicotic studies which gave a resulting estimate of 1.78 (95 % CI 1.07–2.96) for SMR studies and 1.18 (95 % CI 0.86–1.62) for SIR studies. Between-study heterogeneity was statistically significant in silicotic and non-silicotic studies with SMR as risk measure (I^2^ = 94 % with *p* < 0.0001 and I^2^ = 74 % with *p* = 0.013 respectively) but they were found to be lower and statistically insignificant in silicotic and non-silicotic studies with SIR as measure of association (I^2^ = 25 % with *p* = 0.377 and I^2^ = 41 % with *p* = 0.192) respectively.

Subgroup analyses were carried out individually for SMR and SIR studies. We could not perform similar subgroup analysis for the few remaining studies reporting other measures of association due to their limited number. Since the level of between-study heterogeneity was found to be 0 % in the group of OR studies reporting incidence as outcome, we did not perform any further analysis to explore heterogeneity. Subgroup analysis for SMR studies showed a positive association between silica dust exposure and lung cancer in all subgroups except in the subgroup of cement industries which had a pooled risk estimate of 0.87 (95 % CI 0.42–1.82). Heterogeneity became non-significant (*p* > 0.10) in the subgroups of potteries (I^2^ = 0 % and *p* = 0.273), construction industries (I^2^ = 0 % and p-0.656) and in the subgroup including studies done in Australia (I^2^ = 20 % and *p* = 0.265). In all other subgroups, between-study heterogeneity remained significant. Out of 63 SMR studies, only 2 adjusted for smoking [28, 102] and the effect measure in this subgroup was 1.83 (95 % CI 1.51–2.22). In the subgroup of studies without adjustment for smoking, the pooled estimate was 1.55 (95 % CI 1.37–1.75). Thirteen SMR studies having none of the other potential occupational carcinogens mentioned in the Methods Section yielded an estimate of 1.32 (95 % CI 1.14–1.54). The positive association between silica and lung cancer became weaker with increasing quality of the included studies, from 2.56 (95 % CI 1.57–4.19) among SMR studies with an NOS score of 1–3 to 1.24 (95 % CI 1.01–1.52) in those with an NOS score of 7–9. A similar trend was observed among SIR studies.

Subgroup analysis for SIR studies showed a positive relation between occupational silica exposure and risk of lung cancer in all subgroups, with statistically significant risk estimates in all subgroups except that including studies conducted in Canada (*p* = 0.494). Much of the between-study heterogeneity could be explained by sub-grouping the SIR studies and in most of the subgroups, it became statistically insignificant (*p* > 0.1). None of the SIR studies adjusted for smoking. Detailed main statistical analysis, subgroup analyses for SMR and SIR studies and statistical analysis of silicotic and non-silicotic studies are shown in Table 2.

**Table 2:** Results of meta-analysis of all studies, silicotic studies and non-silicotic studies and subgroup analyses

Study design (effect measure)	Number of studies	Effect estimate	*P* value of effect	*P* value of heterogeneity, Q	I^2^	*P* value Egger test
Cohort studies (SMR)	63	1.55 (1.38–1.75)	5.68E-13	<0.0001	96.18	0.02
Cohort studies (SIR)	19	1.68 (1.45–1.96)	1.36E-11	4.59E-08	74.51	0.24
Cohort studies (RR)	1	1.65 (1.13–2.40)	0.01	1		0.72
Case-control mortality studies (OR)	5	1.82 (1.25–2.66)	0.0017	0.1070	51.17	0.51
Case-control incidence studies (OR)	9	1.34 (1.24–1.46)	<0.0001	0.2075	0	0.46
Case-control studies (MOR)	3	1.69 (1.26–2.26)	<0.0001	<0.0001	86.70	1.00
Proportional mortality studies (PMR)	2	1.10 (0.89–1.36)	0.38	0.10	62.02	1.00
Silicotic studies (SMR)	24	2.32 (1.91–2.81)	<0.0001	<0.0001	94.34	-
Silicotic studies (SIR)	4	2.49 (1.87–3.33)	<0.0001	0.377	25.04	-
Silicotic studies (OR)	3	2.56 (1.84–3.57)	<0.0001	0.345	2.65	-
Silicotic studies (MOR)	3	1.88 (1.31–2.71)	0.0006	<0.0001	86.98	-
Non-silicotic studies (SMR)	4	1.78 (1.07–2.96)	0.027	0.013	74.37	-
Non-silicotic studies (SIR)	2	1.18 (0.86–1.62)	0.292	0.192	41.21	-
Subgroup analysis of SMR studies						
Year of publication						
≤ 1990	16	2.37 (1.76–3.19)	1.24E-08	2.92E-94	95.58	0.35
1991–2000	21	1.44 (1.21–1.71)	2.97E-05	1.25E-30	89.89	0.16
> 2000	26	1.30 (1.16–1.46)	1.05E-05	9.98E-49	93.50	0.60
Industry						
Mine	18	1.48 (1.18–1.86)	0.00	4.73E-59	97.17	0.18
Foundry	4	1.51 (0.99–2.29)	0.05	0.02	86.53	0.75
Pottery	7	1.14 (1.05–1.23)	0.00	0.27	0.02	1.00
Cement	4	0.87 (0.42–1.82)	0.71	<0.0001	84.87	0.75
Construction	2	1.55 (1.31–1.82)	1.94E-07	0.66	0.00	1.00
Stone & granite	8	1.32 (1.15–1.50)	6.24E-05	0.01	65.17	0.72
Mixed	19	2.03 (1.61–2.56)	1.68E-09		96.95	0.73
Country						
Europe	26	1.54 (1.25–1.89)	4.95E-05	4.09E-33	95.70	0.13
USA	15	1.24 (1.12–1.38)	6.24E-05	5.32E-07	79.80	0.06
Canada	5	2.14 (1.46–3.13)	9.27E-05	2.34E-32	95.70	0.82
Australia	2	1.73 (1.51–1.98)	7.65E-15	0.26	19.61	1.00
Asia	14	1.74 (1.27–2.39)	<0.0001		97.56	0.75
Occupational confounders						
Absent	13	1.32 (1.14–1.54)	<0.0001	1.79E-13	87.15	0.06
Present	30	1.35 (1.17–1.57)	7.28E-05	7.37E-55	94.47	0.55
Reported measure adjusted for smoking						
Not adjusted	61	1.55 (1.37–1.75)	4.16E-12		96.37	0.02
Adjusted	2	1.83 (1.51–2.22)	7.23E-10	0.43	0.00	1.00
NOS score						
1–3	6	2.56 (1.57–4.19)	0.00	2.35E-65	96.18	1.00
4–6	35	1.57 (1.36–1.82)	9.02E-10	1.68E-80	93.07	0.45
7–9	15	1.24 (1.01–1.52)	0.042025	1.56E-44	97.23	0.17
Cumulative Silica Dust Exposure (CSDE) (mg/m^3^years)						
0 < CSDE ≤ 0.83	5	1.19 (1.02–1.39)	0.02	0.02	68.92	0.23
0.83 < CSDE ≤ 3.9	5	1.27 (0.89–1.82)	0.19	1.04E-24	97.57	0.48
3.9 < CSDE ≤ 8.35	4	1.33 (0.94–1.87)	0.10	2.97E-10	91.94	0.75
CSDE > 8.35	5	1.36 (0.87–2.13)	0.18	1.15E-21	96.33	0.82
Subgroup analysis of SIR studies						
Year of publication						
≤ 1990	4	2.32 (1.50–3.58)	<0.0001	0.07	64.63	0.75
1991–2000	6	1.77 (1.17–2.69)	0.01	5.07E-09	85.81	0.47
> 2000	9	1.54 (1.40–1.70)	8.29E-18	0.41	17.30	1.00
Industry						
Mine	2	1.67 (1.32–2.13)	2.55E-05	0.07	69.32	1.00
Foundry	4	1.40 (1.23–1.58)	2.67E-07	0.52	7.15	0.33
Pottery	1	2.34 (0.62–8.84)	0.21	1		0.33
Cement	3	1.34 (1.01–1.76)	0.04	0.43	12.61	1.00
Construction	1	1.50 (1.26–1.79)	5.04E-06	1		1.00
Granite	3	1.94 (1.55–2.44)	1.33E-08	0.93	0.00	1.00
Mixed	5	2.13 (1.18–3.87)	0.01	4.08E-05	85.96	0.82
Country						
Europe	13	1.78 (1.48–2.14)	1.16E-09	9.18E-08	77.40	0.13
Canada	2	1.42 (0.52–3.93)	0.49	0.05	74.77	1.00
Australia	1	1.89 (1.57–2.28)	2.26E-11	1		1.00
Asia	3	1.38 (1.10–1.73)	<0.0001	0.28	6.72	1.00
Occupational confounders						
Absent	3	1.94 (1.55–2.44)	1.33E-08	0.93	0.00	1.00
Present	12	1.57 (1.32–1.87)	4.14E-07	4.45E-08	80.28	0.64
NOS grading						
1–3	3	1.99 (1.19–3.30)	0.01	0.95	0.00	1.00
4–6	11	1.55 (1.28–1.87)	5.74E-06	2.36E-08	82.02	0.76
7–9	1	1.61 (1.21–2.14)	<0.001	1		0.76

*I* ^*2*^ variability due to heterogeneity; R^2^, *SMR* standardized mortality ratio, *SIR* standardized incidence ratio *OR* odds ratio, *MOR* mortality odds ratio, *PMR* proportional mortality ratio, *NOS* Newcastle-Ottawa scale

Separate meta-regression analyses were performed for SMR studies, SIR studies and silicotic studies reporting SMR as risk measure. These were done using both univariate and multivariate models. In the univariate meta-regression analysis of SMR studies, NOS score was the most important covariate accounting for 19 % of heterogeneity while in the multivariate analysis, the combination of industrial setting, year of publication, geographical location and number of subjects accounted for the maximum amount of heterogeneity (R^2^ = 37 %). As for the SIR studies, univariate analysis showed that total number of deaths accounted for the highest amount of heterogeneity (R^2^ = 100 %) and a combination of number of subjects and NOS score corrected the maximum amount of heterogeneity (R^2^ = 15 %) in multivariate analysis. Regarding the silicotic studies, the combination of year of publication and total number of deaths corrected 43 % of between-study heterogeneity. Detailed results of meta-regression analyses are shown in Table 3.

**Table 3:** Results of meta-regression analyses

Measure	Parameter	k	Estimate	*P* value estimate	*P* value of Q	I^2^	R^2^	*p* covariates
Meta-regression analysis of SMR studies								
SMR	No covariate	63	1.55 (1.38–1.75)	<0.0001	<0.0001	96.18		
Univariate model	Year of publication	63	2.30E + 18 (2.41E + 08–2.19E + 28)	0.003	<0.0001	95.44	15.12	0.000
	Industry	63	1.15 (0.86–1.53)	0.353	<0.0001	95.71	8.15	0.027
	Person-years of follow-up	22	1.53 (1.25–1.87)	<0.0001	<0.0001	94.73	13.4	0.050
	NOS score	63	3.53 (2.27–5.49)	<0.0001	<0.0001	95.11	19.18	0.000
	Number of subjects	63	1.69 (1.48–1.92)	<0.0001	<0.0001	95.43	10.89	0.008
	Total number of deaths	54	1.69 (1.48–1.94)	<0.0001	<0.0001	95.31	13.94	0.006
Multivariate model	Industry, year of publication	63	1.07E + 19 (3.85E + 09-2.97E + 28)	<0.0001	<0.0001	94.65	25.51	<0.0001
	Industry, year of publication, geographical location	63	1.04E + 20 (4.90E + 10-2.21E + 29)	<0.0001	<0.0001	94.3	28.85	<0.0001
	Industry, year of publication, geographical location, number of subjects	63	1.80E + 17 (1.67E + 08-1.94E + 26)	<0.0001	<0.0001	93.18	37.41	<0.0001
	Industry, year of publication, geographical location, number of subjects, NOS score	63	1.06E + 16 (5.27E + 06-2.12E + 25)	0.001	<0.0001	93.05	37.04	<0.0001
	Industry, year of publication, geographical location, number of subjects, total deaths	54	5.32E + 18 (1.07E + 08-2.65E + 29)	0.001	<0.0001	92.72	34.76	<0.0001
	Industry, year of publication, geographical location, NOS score	63	1.29E + 17 (3.61E + 07-4.61E + 26)	<0.0001	<0.0001	93.77	32.23	<0.0001
	Person-years of follow-up, industry	22	1.22 (0.76–1.96)	0.408	<0.0001	94.12	13.87	0.086
	Person-years of follow-up, industry, year of publication	22	2.80E + 14 (2.88E-08-2.73E + 36)	0.198	<0.0001	92.81	17.99	0.079
	Person-years of follow-up, industry, year of publication, geographical location	22	1.55E + 23 (4.66E-02-5.12E + 47)	0.064	<0.0001	92.31	23.08	0.056
	Person-years of follow-up, number of subjects	22	1.62 (1.31–2.00)	<0.0001	<0.0001	92.9	17.36	0.055
Meta-regression analysis of SIR studies								
SIR	No covariate	19	1.68 (1.45–1.96)	<0.0001	<0.0001	74.51		
Univariate model	Year of publication	19	7.92E + 11 (3.71E-02-1.69E + 25)	0.080	<0.0001	72.31	4.27	0.086
	Industry	19	1.37 (0.97–1.95)	0.077	<0.0001	70.88	11.68	0.214
	Number of subjects	19	1.88 (1.55–2.28)	<0.0001	<0.0001	69.94	18.16	0.086
	Total number of deaths	5	1.29 (1.05–1.59)	0.017	0.687	0	100	0.017
Multivariate model	Number of subjects, exposure level	19	2.09 (1.47–2.96)	<0.0001	<0.0001	72.12	9.02	0.197
	Number of subjects, NOS score	19	3.01 (1.52–5.95)	0.002	<0.0001	69.36	15.16	0.087
	Number of subjects, industry	19	1.71 (1.03–2.85)	0.037	<0.0001	70.28	10.84	0.229
Meta- regression of silicotic studies								
SMR	Year of publication	24	7.03E + 21 (5.05E + 04-9.79E + 38)	0.013	<0.0001	92.23	20.55	0.014
	Year of publication, total number of deaths	20	1.59E + 23 (1.60E + 05-1.57E + 41)	0.012	<0.0001	86.98	42.87	0.002
	Year of publication, total number of deaths, geographical location, industry	20	4.61E + 22 (2.40E + 03-8.85E + 41)	0.021	<0.0001	86.9	33.84	0.022
	Year of publication, total number of deaths, industry	20	6.07E + 22 (1.43E + 04-2.58E + 41)	0.017	<0.0001	87.46	38.8	0.002
	Year of publication, total number of deaths, geographical location	20	9.89E + 22 (2.24E + 04-4.37E + 41)	0.016	<0.0001	86.65	38.3	0.007
	Year of publication, geographical location	24	1.26E + 22 (3.90E + 04-4.08E + 39)	0.013	<0.0001	92.05	16.98	0.050

*k* number of studies, *Q* chi-square test for heterogeneity, *I* ^*2*^ variability due to heterogeneity, *R* ^*2*^ amount of heterogeneity accounted for, *SMR* standardized mortality ratio, *SIR* standardized incidence ratio, *NOS* Newcastle-Ottawa scale

For the exposure-response analysis of the relationship between silica dust and risk of lung cancer, reference was made to the subgroup analysis of SMR studies by average level of cumulative silica dust exposure. Nineteen studies were included. It was found that the risk of lung cancer increased with rising exposure level (risk estimate rose from 1.19 (95 % CI 1.02–1.39) in the first quartile to 1.36 (95 % CI 0.87–2.13) in the fourth quartile). However, the *p* value of the estimate was statistically insignificant for the second, third and fourth quartiles (*p* > 0.05). A high level of between-study heterogeneity was also noted especially with rising quartiles of cumulative silica dust (*p* < 0.0001).

Sensitivity analyses showed that omission of any study did not significantly influence the pooled estimates.

## Discussion

The present meta-analysis, which combines the results from 85 different studies, supports the carcinogenicity of respirable crystalline silica dust on the lung. This positive trend was observed independent of the measure of association and of the level of heterogeneity. The pooled risk estimates in the silicotic studies, which were 2.32 (95 % CI 1.91–2.81) for SMR studies and 2.49 (95 % CI 1.87–3.33) for SIR studies, were found to be higher than those in non-silicotic studies, which were 1.78 (95 % CI 1.07–2.96) for SMR studies and 1.18 (95 % CI 0.86–1.62) for SIR studies. Both silicotic and non-silicotic studies include subjects who are exposed to silica dust. Our results support the hypothesis that silicosis has a stronger association with lung cancer morbidity and mortality than silica exposure on its own. The positive association between silica dust and lung cancer in non-silicotic subjects could probably be due to genetic factors which predispose these individuals to lung cancer with only a minimal exposure to silica dust.

Previous meta-analyses have found a positive association between crystalline silica dust and lung cancer in silicotics and silica-exposed workers, but in non-silicotics, the association was either negative or weakly positive [16, 106–111]. In these published studies, the cohort study subgroups gave pooled estimates ranging from 1.25 (95 % CI 1.18–1.33) to 1.29 (95 % CI 1.20–1.40) in silica-exposed participants, 1.69 (95 % CI 1.32–2.16) to 2.78 (95 % CI 2.41–3.22) in silicotics and 1.19 (95 % CI 0.87–1.57) to 1.20 (95 % CI 1.10–1.30) in non-silicotics. The case-control study subgroups yielded risk estimates ranging from 1.41 (95 % CI 1.18–1.70) to 1.42 (95 % CI 1.22–1.65) in silica-exposed workers, 1.70 (95 % CI 1.15–2.52) to 3.27 (95 % 1.32–8.20) in silicotics and from 0.97 (95 % CI 0.68–1.38) to 1.00 (95 % CI 0.70–1.30) in non-silicotics [13].

Based on the year of publication, we observed a gradual decline in the pooled risk estimate with time from a pooled SMR of 2.37 (95 % CI 1.76–3.19) and a pooled SIR of 2.32 (95 % CI 1.50–3.58) in publications before 1991 to a pooled SMR of 1.30 (95 % CI 1.16–1.46) and a pooled SIR of 1.54 (95 % CI 1.40–1.70) in papers published after 2000. Though some of the papers are updates of older ones, the difference between them is the extended follow-up period in the more recent ones. The lowering risk of lung cancer in recent years may be due to more objective outcome assessment and exposure ascertainment by direct measurement and also due to lower dust concentration as a result of the improvement and stricter implementation of dust control measures.

Our study also showed that the risk of lung cancer differed among various industries. In the SMR studies, the highest pooled risk estimate of 1.48 (95 % CI 1.18–1.86) which was statistically significant was observed in the mining industry. Possible reasons may be due to the higher level of silica exposure and longer duration of time spent in dust-laden environment. In the same subgroup, the lowest risk of lung cancer was observed in the pottery factories with a risk estimate of 1.14 (95 % CI 1.05–1.23). This may be because clay coatings decrease the biological availability of the toxic crystalline silica surfaces, thereby diminishing or deferring the disease risk. Harrison et al. found that the percentage of clay coating silica particles was 45 % in pottery worksites, 18 % in tin mines and 13 % in tungsten mines [112]. Studies have shown that clay and aluminum oxide or aluminosilicate surface coatings of respirable crystalline silica particle surfaces can modify the cytotoxic and fibrogenic activities of crystalline silica dust [113]. A negative association, with a risk estimate of 0.87 (95 % CI 0.42–1.82), without statistical significance (*p* = 0.714) was observed between cement dust exposure and lung cancer mortality in cement factory workers in this meta-analysis. A reverse trend was observed among SIR studies in the subgroup of cement industries. Possible explanations could be that cement dust is weakly carcinogenic and cement factory workers are immediately removed from the high-risk job as soon as they are diagnosed with any respiratory problems, thereby decreasing the chance of progress to more severe disease and mortality.

When conducting a meta-analysis of epidemiological studies, significant heterogeneity in risk across studies reflects differences in workplace exposures, assessment of exposure, data collection processes, population being studied and, in the case of silica, in the biological activity of the silica particles. It has been suggested that a variable biological activity of silica particulates might be related to particle size, time since fracture and presence of other minerals or dust components that might cover the silenol radicals on the surface of the silica particles [114]. We were able to explore sources of heterogeneity to varying extent in our study through meta-regression analysis (up to 100 % heterogeneity could be corrected when total number of deaths was used as covariate in the univariate analysis of SIR studies). Higgins commented that heterogeneity is an inevitable part of a meta-analysis and that any amount of heterogeneity is acceptable, provided the predefined eligibility criteria for the meta-analysis are sound and the data are correct, both of which have been duly verified in this study [18].

The exposure-response analysis showed that the higher the level of cumulative silica dust exposure, the more is the risk of lung cancer. However, the high level of heterogeneity limits any inference of causality. Wrong estimation of the level and duration of exposure of workers, varying measurement methods and incorrect data collection may lead to significant between-study heterogeneity in the determination of an exposure-response relationship. Similar findings were obtained in the dose-response meta-analysis of silica and lung cancer using 4 cohort and 6 case-control studies performed by Lacasse et al. [16].

The first strength of our meta-analysis is that we have tried to include the maximum number of relevant studies published till date. The number and variety of studies included in a meta-analysis are sometimes reduced to increase the homogeneity of the studies evaluated. However, this potentially reduces the amount of information on factors that influence the outcome of individual studies. To our knowledge, our meta-analysis is the largest one conducted on this topic. Secondly, we conformed to the PRISMA guidelines for a systematic and objective data analysis. Thirdly, subgroup and meta-regression analyses have allowed us to explore in more detail the issue of heterogeneity which, as expected was substantially high. Fourthly, we have been able to explore the exposure-response relationship between occupational exposure to silica dust and risk of lung cancer.

Two main limitations of our study should be noted. First is the ensuing risk of bias of the included studies. Although publication bias was not detected from funnel plots and by Egger’s regression test of OR and SIR studies, it was found to be significant (*p* < 0.05) for SMR studies. The most important factors that can account for confounding bias in the interpretation of the results are cigarette smoking and occupational carcinogens including radon, arsenic, PAH, diesel, talc, cadmium and asbestiform fibers. When we compared the pooled risk estimate of smoking-adjusted SMR studies with that of the unadjusted studies, we found that cigarette smoking does not account for increased risk of lung cancer among silica-exposed workers. We obtained similar results by subdividing SMR studies into those with potential exposure to occupational confounders and those excluding their presence. These findings imply that the presence of other potential lung carcinogens in silica-exposed jobs does not suggest a confounding effect on the positive relationship between silica and lung cancer. Observational studies are also prone to biases due to selection of study population and loss to follow-up. It is, however difficult to completely control or eliminate all bias when designing or performing an observational study [115]. Our meta-analysis has made an attempt to address this limitation by conducting subgroup analysis based on NOS score. We found that the lower-quality studies tend to overestimate the effect measure, probably due to reliance on self-reporting rather than objective assessment of outcome and indirect methods of measurement of past exposure to silica dust among workers. Self-reporting are usually subject to recall bias leading to exposure and outcome misclassification and overestimation of risk estimates. Second drawback is the high degree of between-study heterogeneity noted except in the group of case-control studies with incidence as outcome.

We have shown, through this meta-analysis that the risk of lung cancer is higher in workers exposed to crystalline silica dust but the exact mechanism of carcinogenicity in human beings are yet to be determined. Three mechanisms have been proposed based on experimental studies in animals. First, exposure to crystalline silica impairs alveolar-macrophage-mediated particle clearance thereby increasing persistence of silica in the lungs, which results in macrophage activation, and the sustained release of chemokines and cytokines. In rats, persistent inflammation is characterized by neutrophils that generate oxidants that induce genotoxicity, injury and proliferation of lung epithelial cells leading to the development of lung cancer. Second, extracellular generation of free radicals by crystalline silica depletes antioxidants in the lung-lining fluid. Third, crystalline silica particles are taken up by epithelial cells followed by intracellular generation of free radicals that directly induce genotoxicity. The IARC considers the first mechanism as the most prominent based on the current experimental data using inhalation or intratracheal instillation in rats, although the other mechanisms cannot be excluded. More research has been recommended in this particular field [13].

## Conclusion

To conclude, this paper supports the positive association of crystalline silica and lung cancer and the existence of an exposure-response relationship between these two, with a high degree of heterogeneity in the analyses. The risk tends to be more pronounced in the presence of silicosis and in the mining industry and is not significantly affected by the presence or exclusion of occupational confounding factors or by adjustment for cigarette smoking. A gradual reduction in the risk with time has also been noted. Further research is needed to find out whether non-silicotics are truly at risk, whether a predisposing factor would explain this potential risk and to determine the mechanism of carcinogenicity of silica in humans.

## Additional files

Additional file 1: Search strategy for Medline database. (DOC 36 kb)
Additional file 2: Characteristics of all studies included. (DOC 232 kb)
Additional file 3 Newcastle-Ottawa Scale (NOS) score for cohort and case-control studies. (DOC 141 kb)
Additional file 4: Funnel plot for studies with standardized mortality ratio (SMR) as measure of association. (DOC 107 kb)
Additional file 5: Funnel plot for studies with standardized incidence ratio (SIR) as measure of association. (DOC 109 kb)
Additional file 6: Funnel plot for studies with odds ratio (OR) as measure of association. (DOC 144 kb)

## Abbreviations

CI: Confidence interval
IARC: International Agency for Research on Cancer
MOR: Mortality odds ratio
NOS: Newcastle-Ottawa scale
OR: Odds ratio
PAH: Polycyclic aromatic hydrocarbons
PMR: Proportional mortality ratio
PRISMA: Preferred reporting items for systematic reviews and meta-analyses
SE: Standard error
SIR: Standardized incidence ratio
SMR: Standardized mortality ratio
