# Supplementary material for: Occupational exposure to silica dust and risk of lung cancer: an updated meta-analysis of epidemiological studies
Source: BMC Public Health. 2016 Nov 4;16:1137. doi: 10.1186/s12889-016-3791-5 (PMC5095988; doi:10.1186/s12889-016-3791-5)
Supplement: Additional file 1: — Search strategy for Medline database. (DOC 36 kb) [file 12889_2016_3791_MOESM1_ESM.doc]

**Additional file 2: Search strategy for Medline database on 29/04/2016**

| Index | Search terms | number of records |
| --- | --- | --- |
| 1 | (cancer occupation* silica OR silicosis exposure) AND (risk incidence mortality) | 108 |
| 2 | cancer occupation silica | 330 |
| 3 | cancer occupation silica (risk incidence mortality) | 51 |
| 4 | cancer occupation silica (risk OR incidence OR mortality) | 290 |
| 5 | ("cancer" OR "neoplasm*") AND ("occupation*" OR "work*"silica) AND ("risk" OR "incidence" OR "mortality" OR "survival") | 381 |
| 6 | ("cancer" OR "neoplasm*") AND ("occupation*" OR "work*") AND "silica*" | 272 |
| 7 | cancer AND (occupation* OR work*) AND silica | 3605 |
| 8 | silica cancer (filter used: clinical /observational study) | 215 |
| 19 | silica cancer AND ("study") | 2826 |
| 10 | silica lung risk | 1669 |
| 11 | silica lung cancer risk assessment | 282 |
| 12 | 12 no review and 21 systematic review | 188 |
| 13 | silica lung cancer risk assessment NOT rat NOT asbestos | 68 |
| 14 | ((silica lung cancer risk assessment NOT rat NOT asbestos)) NOT review[Title] | 66 |
| 15 | (((cancer occupation* silica OR silicosis exposure) AND (risk incidence mortality))) NOT review NOT rat | 94 |
| 16 | (lung cancer) AND (silica OR silicosis) AND (risk incidence mortality) | 468 |
| 17 | (lung cancer) AND (silica OR silicosis) AND (risk incidence mortality) NOT asbestos | 146 |
