# Supplementary material for: Occupational exposure to silica dust and risk of lung cancer: an updated meta-analysis of epidemiological studies
Source: BMC Public Health. 2016 Nov 4;16:1137. doi: 10.1186/s12889-016-3791-5 (PMC5095988; doi:10.1186/s12889-016-3791-5)
Supplement: Additional file 2: — Characteristics of all studies included. (DOC 232 kb) [file 12889_2016_3791_MOESM2_ESM.doc]

# Additional file 3 Characteristics of all studies included

|  | **Author, Year, Subgroup** | **N** | **Total deaths** | **Country** | **Factory** | **Covariates adjusted for** | **Smoking-adjusted** | **Measure** | **Effect estimate (95% CI)** | **Lung cancer deaths** | **Follow-up period** | **PY** |
| --- | --- | --- | --- | --- | --- | --- | --- | --- | --- | --- | --- | --- |
|  | **Cohort Studies** |  |  |  |  |  |  |  |  |  |  |  |
| 1 | Ahlman, 1991 [1] | 597 | 102 | Finland | Mine | Age, sex | No | SMR | 1.45 (0.70 -2.70) | 10 | 1954-1986 | . |
| 2 | Ahn , 2010 [2] | 17098 |  | Korea | Foundry | Age,sex,calendar period | No | SIR | 1.45 (1.11-1.87) | 61 | 1992-2005 | . |
| 3 | Amandus, 1995 [3] | 760 | 550 | USA | Mixed | Age, sex,race,talc,asbestos | No | SMR | 2.30 (1.50 -3.40) |  | 1940-1983 | . |
| 4 | Andjelkovich, 1994 [4], white | 5337 | 1695 | USA | Foundry | Age,sex,race | No | SMR | 1.23 (0.96-1.54) | 72 | 1950-1989 | . |
| 5 | Andjelkovich, 1994 [4], non-white | 3437 |  | USA | Foundry  (others) | Age,sex,race | No | SMR | 1.32 (1.02-1.67) | 67 | 1950-1989 | 36890 |
| 6 | Bergdahl , 2010 [5] | 8321 |  | Sweden | Iron mine | Age,sex,calendar period | No | SIR | 1.48 (1.22-1.78) | 112 | 1958-2000 | 227000 |
| 7 | Berry, 2004 [6] | 1447 | 1078 | Australia | Mixed | Age,sex,calendar period,smoking | Yes | SMR | 1.90 (1.50-2.30) | 94 | 1968-2000 | 101177 |
| 8 | Brown, 2005 [7] | 2703 | 764 | UK | Sand | Age,sex,calendar period | No | SMR | 0.99 (0.78-1.24) | 77 | 1950-2001 | 26113 |
| 9 | Carta, 2001 [8] | 724 | 579 | Sardinia | Mine & quarries | Age,sex,calendar period | No | SMR | 1.37 (0.98-1.91) | 34 | 1961-1987 | 269253 |
| 10 | Chan, 2000 [9] | 1502 | 286 | Hong Kong | Mixed | Age,sex,calendar period | No | SMR | 1.94 (1.35- 2.70) | 33 | 1981-1997 | . |
| 11 | Chen, 2012 [10] | 74040 | 19516 | China | Mine & pottery | Age,sex,calendar period | No | SMR | 0.90 (0.84-0.97) | 949 | 1974-2003 | 128970.2 |
| 12 | Chen, 2006 [11] | 7837 | 1094 | China | Mine | Age, sex | No | SMR | 2.49 (2.09-2.94) | 138 | 1972-1994 | . |
| 13 | Chen, 1992, Pottery [12] | 9017 | 1592 | China | Pottery | Age,sex | No | SMR | 1.10 (0.84-1.40) | 68 | 1972-1989 | 86561 |
| 14 | Chen, 1992, Tin mine [12] | 7858 | 956 | China | Tin mine | Age,sex | No | SMR | 2.10 (1.70 -2.60) | 97 | 1972-1989 | 76161.7 |
| 15 | Chen, 1992, Tungsten mine [12] | 28481 | 4549 | China | Tungsten mine | Age,sex | No | SMR | 0.63 (0.53-0.75) | 135 | 1972-1989 | 2306428 |
| 16 | Chen, 1990 [13] | 6444 | 550 | China | Iron mine | Age,sex | No | SMR | 3.70 (2.50-5.30) | 29 | 1970-1989 | 237178 |
| 17 | Cherry, 2013 [14] | 5115 | 1904 | UK | Pottery | Age,sex,calendar period | No | SMR | 1.15 (1.01-1.30) | 243 | 1985-2008 | . |
| 18 | Chia, 1991 [15] | 159 |  | China | Granite | Age,sex,calendar period | No | SIR | 2.01 (0.92-3.81) | 9 | 1970-1984 | 73257 |
| 19 | Chiyotani, 1990 [16] | 1941 | 352 | Japan | Mixed | Age,sex | No | SMR | 6.03 (5.29-6.77) | 44 | 1979-1983 | . |
| 20 | Cocco, 1994 [17] | 4740 | 1205 | Sardinia | Mine | Age,sex,calendar period | No | SMR | 0.95 (0.76-1.17) | 86 | 1960-1988 | 119390.5 |
| 21 | Costello, 1995 [18] | 3246 | 661 | USA | Stone | Age,sex,race,calendar period | No | SMR | 1.29 (0.96-1.70) | 51 | 1940-1980 | 17508.4 |
| 22 | Finkelstein, 1995 [19], silicotics | 328 |  | Canada | Mixed (silicotic) | Age,sex | No | SIR | 2.55 (1.43-8.28) | 15 | 1974-1992 | . |
| 23 | Finkelstein, 1995 [19], non-silicotics | 970 |  | Canada | Mixed (others) | Age,sex | No | SIR | 0.90 (0.51-1.47) | 16 | 1974-1992 | 26976 |
| 24 | Finkelstein, 2005 [20] | 10953 | 836 | Canada | Construction | Age,sex,calendar period | No | SMR | 1.58 (1.30-1.90) | 126 | 1950-1970 | . |
| 25 | Finkelstein, 1982 [21], Mine | 1190 | 905 | Canada | Mine | Age,sex,calendar period | No | SMR | 2.30 (1.80-3.00) | 62 | 1940-1975 | . |
| 26 | Finkelstein, 1982 [21], Surface industry | 289 | 206 | Canada | Surface industry | Age,sex,calendar period | No | SMR | 3.02 (1.70-4.90) | 16 | 1940-1975 | . |
| 27 | Gallagher, 2015 [22] | 2342 | 1219 | USA | DE | Age,sex,race,calendar period | No | SMR | 1.03 (0.85-1.23) | 113 | 1942-2011 | . |
| 28 | Giordano, 2011 [23] | 748 | 280 | Italy | Cement | Age,sex,calendar period | No | SMR | 0.29 (0.13-0.58) | 8 | 1956-2006 | . |
| 29 | Goldsmith, 1995 [24] | 590 | 421 | USA | Mixed | Age,sex,calendar period | No | SMR | 1.90 (1.35-2.60) | 39 | 1945-1991 | . |
| 30 | Graber, 2014 [25] | 8829 | 5907 | USA | Mine | Age,sex,race,calendar period | No | SMR | 1.08 (1.00-1.18) | 568 | 1969-2007 | . |
| 31 | Graham, 2004 [26] | 5545 | 1762 | USA | Granite | Age,sex,race,calendar period,area | No | SMR | 1.18 (1.03-1.35) | 201 | 1940-1996 | . |
| 32 | Guenel, 1989 [27] | 1081 |  | Denmark | Stone | Age,sex,calendar period | No | SIR | 2.00 (1.49-2.69) | 44 | 1943-1984 | . |
| 33 | Guenel , 1989 [27] | 990 |  | Denmark | Stone | Age,sex,calendar period | No | SIR | 1.81 (1.16-2.70) | 24 | 1943-1984 | . |
| 34 | Hodgson, 1990 [28] | 3010 | 851 | UK | Mine | Age,sex | No | SMR | 1.58 (1.29-1.91) | 105 | 1941-1984 | 29288 |
| 35 | Infante-Rivard, 1989 [29] | 1072 | 565 | Canada | Mixed | Age,sex,calendar period | No | SMR | 3.47 (3.11-3.90) | 83 | 1938-1986 | 14202 |
| 36 | Kauppinen , 2003[30] | 5676 | 666 | Finland | Pavement | Age,sex,calendar period | No | SMR / SIR | 1.45 (1.03-1.98) / 1.45 (1.03-1.98) | 51 , 110 | 1964-1994 | 97915 |
| 37 | Koh, 2011 [31] | 5146 | 103 | Korea | Cement | Age,sex,calendar period | No | SMR | 1.05 (0.68-1.57) | 24 | 1992-2007 | . |
| 38 | Koh, 2011 [31] | 5596 | 174 | Korea | Cement | Age,sex,calendar period | No | SIR | 1.08 (0.70-1.60) | 25 | 1997-2005 | 13003.1 |
| 39 | Koskela , 1994 [32] | 1026 | 363 | Finland | Granite | Age,sex,calendar period | No | SMR | 1.40 (0.98-1.93) | 31 | 1940-1989 | . |
| 40 | Kusiak , 1991 [33] | 13603 |  | Canada | Gold mine | Age,sex | No | SMR | 1.29 (1.15-1.45) | . | 1955-1986 | 52608 |
| 41 | Lawler , 1983 [34], Underground iron mine | 4708 |  | USA | Mine | Age,sex,race | No | SMR | 1.00 (0.82-1.19) | 117 | 1937-1978 | 1666 |
| 42 | Lawler , 1983 [34], Surface iron mine | 5695 |  | USA | Surface industry | Age,sex,race | No | SMR | 0.88 (0.71-1.07) | 95 | 1937-1978 | . |
| 43 | Marinaccio , 2006 [35] | 14929 | 8521 | Italy | Mixed | Age,sex,calendar period | No | SMR | 1.10 (1.03-1.18) | 798 | 1980-1999 | . |
| 44 | Mehnert , 1990 [36] | 2483 | 387 | Germany | Quarry | Age,sex | No | SMR | 1.09 (0.72-1.59) | 27 | 1970-1985 | 70388 |
| 45 | Meijers , 1996 [37] | 1784 |  | Netherlands | Ceramic | Age,sex,calendar period | No | SMR | 0.88 (0.59-1.26) | 30 |  | 183170 |
| 46 | Merlo , 1991 [38] | 1022 | 243 | Italy | Refractory Brick | Age,sex,calendar period | No | SMR | 1.51 (1.00-2.18) | 28 | 1954-1986 | . |
| 47 | Merlo , 1995 [39] | 450 | 290 | Italy | Mixed | Age,sex,calendar period | No | SMR | 3.50 (2.44-4.87) | 35 | 1961-1987 | 14782 |
| 48 | Miller , 2009 [40] | 17820 | 10698 | UK | Mine | Age,sex,calendar period | No | SMR | 0.99 (0.93-1.05) | 958 | 1959-2006 | . |
| 49 | Moulin , 1999 [41] | 4897 | 649 | France | Foundry | Age,sex,calendar period | No | SMR | 1.19 (0.89-1.55) | 54 | 1968-1992 | . |
| 50 | Ng, 1990 [42] | 1419 | 356 | HongKong | Mixed | Age,sex,PAH, asbestos | No | SMR | 2.03 (1.35-2.93) | 28 | 1980-1986 | . |
| 51 | Olsen , 2012 [43] | 2650 | 772 | USA | Mine & mill | Age,sex,race,calendar period | No | SMR | 1.11 (0.88-1.39) | 77 | 1945-2004 | . |
| 52 | Partanen , 1994 [44] | 811 |  | Finland | Mixed | Age,sex,calendar period | No | SIR | 2.89 (2.35-3.48) | 190 | 1936-1977 | . |
| 53 | Peters , 2013 [45] | 2294 | 1922 | Australia | Mine | Age,sex,calendar period | No | SMR / SIR | 1.64 (1.42-1.90) / 1.64 (1.42-1.90) | 138, 110 | 1961-1975 | . |
| 54 | Pham , 1983 [46] | 1173 | 40 | France | Iron mine | Age,sex | No | SMR | 3.50 (1.90-6.00) | 13 |  | . |
| 55 | Preller , 2010 [47] | 58279 |  | Netherlands | mixed | Age,sex,smoking,other confounders | Yes | RR (incidence) | 1.65 (1.14-2.41) | 1667 | 1986-1997 | 21427 |
| 56 | Rafnsson , 1997 [48] | 1342 |  | Iceland | DE | Age,sex,calendar period | No | SIR | 2.34 (0.48-6.85) | 5 | 1968-1984 | . |
| 57 | Reid , 1996 [49] | 4925 | 2032 | South Africa | Gold mine | Age,sex,race | No | SMR | 1.40 (1.18-1.65) | 143 | 1967-1985 | . |
| 58 | Scarselli , 2011 [50] | 2034 | 1258 | Italy | Mixed | Age,sex,calendar period | No | SMR | 1.39 (1.17-1.64) | 139 | 1950-1984 | . |
| 59 | Sherson , 1991 [51] |  | 6144 | Denmark | Foundry | Age,sex,calendar period | No | SIR | 1.30 (1.12-1.51) | 166 | 1967-1985 | . |
| 60 | Smailyte , 2004 [52], male | 1727 | 376 | Lithuania | Cement (male) | Age,sex,calendar period | No | SMR / SIR | 1.40 (1.00-1.90) / 1.40 (1.00-1.90) | 36, 44 | 1978-2000 | . |
| 61 | Smailyte , 2004 [52], female | 771 | 74 | Lithuania | Cement (female) | Age,sex,calendar period | No | SMR / SIR | 1.40 (0.30-5.50) / 1.40 (0.30-5.50) | 2, 3 | 1978-2000 | . |
| 62 | Steenland , 1995 [53] | 3328 | 1551 | USA | Mine | Age,sex | No | SMR | 1.13 (0.94-1.36) | 115 | 1940-1990 | . |
| 63 | Steenland , 2001 [54] | 4626 | 1073 | USA | Sand | Age,sex,calendar period | No | SMR | 1.60 (1.31-1.93) | 109 | 1974-1995 | . |
| 64 | Thomas , 1990 [55] | 2055 | 578 | USA | Ceramic | Age,sex | No | SMR | 1.43 (1.07-1.88) | 52 | 1939-1980 | . |
| 65 | Tornling , 1991 [56] | 280 | 218 | Sweden | Mixed | Age,sex | No | SMR | 2.36 (1.07-4.48) | 9 | 1951-1985 | . |
| 66 | Tse , 2014 [57] | 3202 | 1033 | HongKong | Mixed | Age,sex,calendar period | No | SMR | 1.86 (1.59-2.17) | 157 | 1981-2006 | . |
| 67 | Ulm , 2004 [58] | 440 | 144 | Germany | stone and quarry | Age,calendar period | No | SMR | 2.40 (1.37-3.90) | 16 | 1988-2001 | 2659 |
| 68 | Vacek , 2010 [59] | 7052 | 3845 | USA | Granite | Age,sex,calendar period | No | SMR | 1.37 (1.23-1.52) | 359 | 1947-2004 | . |
| 69 | Wang , 1996 [60] | 4372 | 974 | China | Metallurgy | Age,sex,calendar period | No | SMR | 2.37 (1.96-2.86) | 104 | 1980-1989 | . |
| 70 | Westburg, 2013 [61] | 3045 |  | Sweden | Foundry | Age,sex,calendar period | No | SIR | 1.61 (1.20-2.12) | 52 | 1958-2004 | . |
| 71 | Westerholm, 1980 [62] | 3610 |  | Sweden | Mixed | Age,sex,calendar period | No | SMR | 3.80 (2.30-5.80) |  | 1931-1969 | . |
| 72 | Westerholm, 1986 [63], Mine, quarry, tunnelling | 284 |  | Sweden | Mixed | Age,sex,calendar period | No | SMR / SIR | 5.38 (2.20-11.10) / 5.38 (2.20-11.10) | 7, 9 | 1961-1980 | . |
| 73 | Westerholm, 1986 [63], Foundry | 428 |  | Sweden | Foundry | Age,sex,calendar period | No | SMR / SIR | 3.85 (1.80-7.10) / 3.85 (1.80-7.10) | 10, 6 | 1961-1980 | . |
| 74 | Wiebert, 2014 [64] | 436 |  | Sweden | Mixed | Age,sex,calendar period | No | SIR | 1.39 (0.79-2.25) |  | 1971-2006 | . |
| 75 | Yu, 2008 [65] | 2798 |  | Hong Kong | mixed | Age,calendar period, smoking | Yes | SMR | 1.56 (0.98-2.36) | 86 | 1981-1999 | 24993 |
| 76 | Zambon , 1987 [66] | 1313 | 878 | Italy | Mixed | Age,sex,calendar period | No | SMR | 2.39 (1.86-3.02) | 70 | 1959-1984 | . |
| 77 | Zhang, 2008 [67] | 4851 | 1542 | China | Ceramic | Age,sex,calendar period | No | SMR | 1.12 (0.91-1.37) | 95 | 1972-2003 | 87247 |
|  | **Case-control Studies** |  |  |  |  |  |  |  |  |  |  |  |
| 1 | Bruske-Hohfeld, 2000 [68] | 7039 |  | Germany | Mixed | Age,area,smoking, asbestos | Yes | OR (incidence) | 1.41 (1.22-1.62) | 3498 | 1988-1996 |  |
| 2 | Cassidy, 2007 [69] | 5956 |  | Europe | Mixed | Age, sex, center, smoking, education, insulation dust and wood dust. | Yes | OR (incidence) | 1.37 (1.14-1.65) | 2852 | 1998-2001 | 13202 |
| 3 | Forastiere, 1989 [70] | 952 | 595 | Italy | Mixed | Age,sex,calendar period | No | MOR | 1.50 (1.10-1.90) | 64 | 1969-1984 | . |
| 4 | Fu, 1994 [71] | 267 |  | China | Tin mine | Age,sex,smoking | Yes | OR (incidence) | 2.13 (1.27-3.60) | 79 | 1973-1989 | 106000 |
| 5 | Kachuri, 2013 [72] | 3634 |  | Canada | Mixed | Age,sex,smoking,diesel, asbestos,gasoline | Yes | OR (incidence) | 1.20 (1.00-1.43) | 1681 | 1994-1997 |  |
| 6 | Lagorio , 1990 [73], non-silicotics |  |  | Italy | Pottery (others) | Age,calendar period, smoking | Yes | OR | 1.40 (0.70-2.80) | . | 1968-1984 | . |
| 7 | Lagorio , 1990 [73], silicotics |  |  | Italy | Pottery (silicotic) | Age,calendar period, smoking | Yes | OR | 3.90 (1.80-8.30) | . | 1968-1984 | . |
| 8 | Neuberger , 1988 [74] | 2212 |  | Austria | Mixed | Age,sex,calendar period,area,smoking | Yes | MOR | 1.41 (1.21-1.64) | 182 | 1950-1960 | . |
| 9 | Rodriguez , 2000 [75] | 24400 |  | Spain | foundry | Age,smoking | Yes | OR (incidence) | 2.55 (1.25-5.21) | 144 | 1952-1995 | . |
| 10 | Samet, 1994 [76] | 281 |  | USA | Uranium mine | Radon | No | OR (incidence) | 1.16 (0.35-3.84) |  | 1950-1984 | . |
| 11 | Schuller , 1986 [77] | 2399 |  | Switzerland | Mixed | Calendar period | No | MOR | 2.23 (1.90-2.60) | 180 | 1987-1993 | . |
| 12 | Tse, 2012 [78] | 2227 |  | China | Mixed | Age,radon,smoking | Yes | OR (incidence) | 1.37 (1.00-1.89) | 1208 | 2004-2006 |  |
| 13 | Tsuda, 2002 [79] | 501 |  | Japan | Mixed | Age,sex,smoking | Yes | OR | 2.06 (1.29-3.29) | 184 | 1986-1993 |  |
| 14 | Ulm, 1999 [80] | 1042 |  | Germany | Mixed | Age,sex,area,smoking | Yes | OR (incidence) | 0.91 (0.57-1.46) | 247 | 1980-1994 |  |
| 15 | Vida, 2010 [81] | 4376 |  | Canada | Mixed | Age,sex,race,smoking | Yes | OR (incidence) | 1.31 (1.08-1.59) | 1595 | 1979-2001 |  |
| 16 | Watkins , 2002 [82] | 172 |  | USA | asphalt roofing | Age,calendar period | No | OR | 1.53 (0.19- 10.76) | 39 | 1977-1997 | . |
| 17 | Xu , 1996 [83] | 1569 |  | China | Foundry | Age,sex,smoking | Yes | OR | 1.40 (1.10-1.80) | 418 | 1980-1989 | . |
|  | **Proportional Mortality Studies** |  |  |  |  |  |  |  |  |  |  |  |
| 1 | Kinlen , 1988 [84] | 1947 | 1604 | UK | Iron mine | Age,sex,calendar period, area | No | PMR | 0.97 (0.77-1.20) | 84 | 1939-1982 | 74123 |
| 2 | Thomas , 1982 [85] | 3870 |  | USA | Ceramic | Age,sex | No | PMR | 1.21 (1.04-1.40) | 178 | 1955-1977 |  |

N, Number of subjects; CI, Confidence interval; PY, Person-years of follow-up; UK, United Kingdom; USA, United States of America; DE, Diatomaceous earth; SMR, Standardized mortality ratio; SIR, Standardized incidence ratio; OR, Odds ratio; MOR, Mortality odds ratio; PMR, Proportional mortality ratio

# References for included studies

1. Ahlman K, Koskela RS, Kuikka P, Koponen M, Annanmaki M: Mortality among sulfide ore miners. American journal of industrial medicine 1991, 19(5):603-617.
2. Ahn YS, Won JU, Park RM: Cancer morbidity of foundry workers in Korea. Journal of Korean medical science 2010, 25(12):1733-1741.
3. Amandus HE, Shy C, Castellan RM, Blair A, Heineman EF: Silicosis and lung cancer among workers in North Carolina dusty trades. Scandinavian journal of work, environment & health 1995, 21 Suppl 2:81-83.
4. Andjelkovich DA, Shy CM, Brown MH, Janszen DB, Levine RJ, Richardson RB: Mortality of iron foundry workers. III. Lung cancer case-control study. Journal of occupational medicine : official publication of the Industrial Medical Association 1994, 36(12):1301-1309.
5. Bergdahl IA, Jonsson H, Eriksson K, Damber L, Jarvholm B: Lung cancer and exposure to quartz and diesel exhaust in Swedish iron ore miners with concurrent exposure to radon. Occupational and environmental medicine 2010, 67(8):513-518.
6. Berry G, Rogers A, Yeung P: Silicosis and lung cancer: a mortality study of compensated men with silicosis in New South Wales, Australia. Occupational medicine (Oxford, England) 2004, 54(6):387-394.
7. Brown TP, Rushton L: Mortality in the UK industrial silica sand industry: 1. Assessment of exposure to respirable crystalline silica. Occupational and environmental medicine 2005, 62(7):442-445.
8. Carta P, Aru G, Manca P: Mortality from lung cancer among silicotic patients in Sardinia: an update study with 10 more years of follow up. Occupational and environmental medicine 2001, 58(12):786-793.
9. Chan CK, Leung CC, Tam CM, Yu TS, Wong TW: Lung cancer mortality among a cohort of men in a silicotic register. Journal of occupational and environmental medicine / American College of Occupational and Environmental Medicine 2000, 42(1):69-75.
10. Chen W, Liu Y, Wang H, Hnizdo E, Sun Y, Su L, Zhang X, Weng S, Bochmann F, Hearl FJ et al: Long-term exposure to silica dust and risk of total and cause-specific mortality in Chinese workers: a cohort study. PLoS medicine 2012, 9(4):e1001206.
11. Chen W, Yang J, Chen J, Bruch J: Exposures to silica mixed dust and cohort mortality study in tin mines: exposure-response analysis and risk assessment of lung cancer. American journal of industrial medicine 2006, 49(2):67-76.
12. Chen J, McLaughlin JK, Zhang JY, Stone BJ, Luo J, Chen RA, Dosemeci M, Rexing SH, Wu Z, Hearl FJ et al: Mortality among dust-exposed Chinese mine and pottery workers. Journal of occupational medicine : official publication of the Industrial Medical Association 1992, 34(3):311-316.
13. Chen SY, Hayes RB, Liang SR, Li QG, Stewart PA, Blair A: Mortality experience of haematite mine workers in China. British journal of industrial medicine 1990, 47(3):175-181.
14. Cherry N, Harris J, McDonald C, Turner S, Taylor TN, Cullinan P: Mortality in a cohort of Staffordshire pottery workers: follow-up to December 2008. Occupational and environmental medicine 2013, 70(3):149-155.
15. Chia SE, Chia KS, Phoon WH, Lee HP: Silicosis and lung cancer among Chinese granite workers. Scandinavian journal of work, environment & health 1991, 17(3):170-174.
16. Chiyotani K, Saito K, Okubo T, Takahashi K: Lung cancer risk among pneumoconiosis patients in Japan, with special reference to silicotics. IARC scientific publications 1990(97):95-104.
17. Cocco PL, Carta P, Belli S, Picchiri GF, Flore MV: Mortality of Sardinian lead and zinc miners: 1960-88. Occupational and environmental medicine 1994, 51(10):674-682.
18. Costello J, Castellan RM, Swecker GS, Kullman GJ: Mortality of a cohort of U.S. workers employed in the crushed stone industry, 1940-1980. American journal of industrial medicine 1995, 27(5):625-640.
19. Finkelstein MM: Radiographic abnormalities and the risk of lung cancer among workers exposed to silica dust in Ontario. CMAJ : Canadian Medical Association journal = journal de l'Association medicale canadienne 1995, 152(1):37-43.
20. Finkelstein MM, Verma DK: Mortality among Ontario members of the International Union of Bricklayers and Allied Craftworkers. American journal of industrial medicine 2005, 47(1):4-9.
21. Finkelstein M, Kusiak R, Suranyi G: Mortality among miners receiving workmen's compensation for silicosis in Ontario: 1940-1975. Journal of occupational medicine : official publication of the Industrial Medical Association 1982, 24(9):663-667.
22. Gallagher LG, Park RM, Checkoway H: Extended follow-up of lung cancer and non-malignant respiratory disease mortality among California diatomaceous earth workers. Occupational and environmental medicine 2015, 72(5):360-365.
23. Giordano F, Dell'orco V, Fantini F, Grippo F, Perretta V, Testa A, Figa-Talamanca I: Mortality in a cohort of cement workers in a plant of Central Italy. International archives of occupational and environmental health 2012, 85(4):373-379.
24. Goldsmith DF, Beaumont JJ, Morrin LA, Schenker MB: Respiratory cancer and other chronic disease mortality among silicotics in California. American journal of industrial medicine 1995, 28(4):459-467.
25. Graber JM, Stayner LT, Cohen RA, Conroy LM, Attfield MD: Respiratory disease mortality among US coal miners; results after 37 years of follow-up. Occupational and environmental medicine 2014, 71(1):30-39.
26. Graham WG, Costello J, Vacek PM: Vermont granite mortality study: an update with an emphasis on lung cancer. Journal of occupational and environmental medicine / American College of Occupational and Environmental Medicine 2004, 46(5):459-466.
27. Guenel P, Hojberg G, Lynge E: Cancer incidence among Danish stone workers. Scandinavian journal of work, environment & health 1989, 15(4):265-270.
28. Hodgson JT, Jones RD: Mortality of a cohort of tin miners 1941-86. British journal of industrial medicine 1990, 47(10):665-676.
29. Infante-Rivard C, Armstrong B, Petitclerc M, Cloutier LG, Theriault G: Lung cancer mortality and silicosis in Quebec, 1938-85. Lancet (London, England) 1989, 2(8678-8679):1504-1507.
30. Kauppinen T, Heikkila P, Partanen T, Virtanen SV, Pukkala E, Ylostalo P, Burstyn I, Ferro G, Boffetta P: Mortality and cancer incidence of workers in Finnish road paving companies. American journal of industrial medicine 2003, 43(1):49-57.
31. Koh DH, Kim TW, Jang SH, Ryu HW: Cancer mortality and incidence in cement industry workers in Korea. Safety and health at work 2011, 2(3):243-249.
32. Koskela RS, Klockars M, Laurent H, Holopainen M: Silica dust exposure and lung cancer. Scandinavian journal of work, environment & health 1994, 20(6):407-416.
33. Kusiak RA, Springer J, Ritchie AC, Muller J: Carcinoma of the lung in Ontario gold miners: possible aetiological factors. British journal of industrial medicine 1991, 48(12):808-817.
34. Lawler AB, Mandel JS, Schuman LM, Lubin JH: A retrospective cohort mortality study of iron ore (hematite) miners in Minnesota. Journal of occupational medicine : official publication of the Industrial Medical Association 1985, 27(7):507-517.
35. Marinaccio A, Scarselli A, Gorini G, Chellini E, Mastrantonio M, Uccelli R, Altavista P, Pirastu R, Merlo DF, Nesti M: Retrospective mortality cohort study of Italian workers compensated for silicosis. Occupational and environmental medicine 2006, 63(11):762-765.
36. Mehnert WH, Staneczek W, Mohner M, Konetzke G, Muller W, Ahlendorf W, Beck B, Winkelmann R, Simonato L: A mortality study of a cohort of slate quarry workers in the German Democratic Republic. IARC scientific publications 1990(97):55-64.
37. Meijers JM, Swaen GM, Slangen JJ: Mortality and lung cancer in ceramic workers in The Netherlands: preliminary results. American journal of industrial medicine 1996, 30(1):26-30.
38. Merlo F, Costantini M, Reggiardo G, Ceppi M, Puntoni R: Lung cancer risk among refractory brick workers exposed to crystalline silica: a retrospective cohort study. Epidemiology (Cambridge, Mass) 1991, 2(4):299-305.
39. Merlo F, Fontana L, Reggiardo G, Ceppi M, Barisione G, Garrone E, Doria M: Mortality among silicotics in Genoa, Italy, from 1961 to 1987. Scandinavian journal of work, environment & health 1995, 21 Suppl 2:77-80.
40. Miller BG, MacCalman L: Cause-specific mortality in British coal workers and exposure to respirable dust and quartz. Occupational and environmental medicine 2010, 67(4):270-276.
41. Moulin JJ, Clavel T, Roy D, Dananche B, Marquis N, Fevotte J, Fontana JM: Risk of lung cancer in workers producing stainless steel and metallic alloys. International archives of occupational and environmental health 2000, 73(3):171-180.
42. Ng TP, Chan SL, Lee J: Mortality of a cohort of men in a silicosis register: further evidence of an association with lung cancer. American journal of industrial medicine 1990, 17(2):163-171.
43. Olsen GW, Andres KL, Johnson RA, Buehrer BD, Holen BM, Morey SZ, Logan PW, Hewett P: Cohort mortality study of roofing granule mine and mill workers. Part II. Epidemiologic analysis, 1945-2004. Journal of occupational and environmental hygiene 2012, 9(4):257-268.
44. Partanen T, Pukkala E, Vainio H, Kurppa K, Koskinen H: Increased incidence of lung and skin cancer in Finnish silicotic patients. Journal of occupational medicine : official publication of the Industrial Medical Association 1994, 36(6):616-622.
45. Peters S, Reid A, Fritschi L, Musk AW, de Klerk N: Cancer incidence and mortality among underground and surface goldminers in Western Australia. British journal of cancer 2013, 108(9):1879-1882.
46. Pham QT, Gaertner M, Mur JM, Braun P, Gabiano M, Sadoul P: Incidence of lung cancer among iron miners. European journal of respiratory diseases 1983, 64(7):534-540.
47. Preller L, van den Bosch LM, van den Brandt PA, Kauppinen T, Goldbohm A: Occupational exposure to silica and lung cancer risk in the Netherlands. Occupational and environmental medicine 2010, 67(10):657-663.
48. Rafnsson V, Gunnarsdottir H: Lung cancer incidence among an Icelandic cohort exposed to diatomaceous earth and cristobalite. Scandinavian journal of work, environment & health 1997, 23(3):187-192.
49. Reid PJ, Sluis-Cremer GK: Mortality of white South African gold miners. Occupational and environmental medicine 1996, 53(1):11-16.
50. Scarselli A, Binazzi A, Forastiere F, Cavariani F, Marinaccio A: Industry and job-specific mortality after occupational exposure to silica dust. Occupational medicine (Oxford, England) 2011, 61(6):422-429.
51. Sherson D, Svane O, Lynge E: Cancer incidence among foundry workers in Denmark. Archives of environmental health 1991, 46(2):75-81.
52. Smailyte G, Kurtinaitis J, Andersen A: Mortality and cancer incidence among Lithuanian cement producing workers. Occupational and environmental medicine 2004, 61(6):529-534.
53. Steenland K, Brown D: Mortality study of gold miners exposed to silica and nonasbestiform amphibole minerals: an update with 14 more years of follow-up. American journal of industrial medicine 1995, 27(2):217-229.
54. Steenland K, Mannetje A, Boffetta P, Stayner L, Attfield M, Chen J, Dosemeci M, DeKlerk N, Hnizdo E, Koskela R et al: Pooled exposure-response analyses and risk assessment for lung cancer in 10 cohorts of silica-exposed workers: an IARC multicentre study. Cancer causes & control : CCC 2001, 12(9):773-784.
55. Thomas TL: Lung cancer mortality among pottery workers in the United States. IARC scientific publications 1990(97):75-81.
56. Tornling G, Hogstedt C, Westerholm P: Lung cancer incidence among Swedish ceramic workers with silicosis. IARC scientific publications 1990(97):113-119.
57. Tse LA, Yu IT, Qiu H, Leung CC: Joint effects of smoking and silicosis on diseases to the lungs. PloS one 2014, 9(8):e104494.
58. Ulm K, Gerein P, Eigenthaler J, Schmidt S, Ehnes H: Silica, silicosis and lung-cancer: results from a cohort study in the stone and quarry industry. International archives of occupational and environmental health 2004, 77(5):313-318.
59. Vacek PM, Verma DK, Graham WG, Callas PW, Gibbs GW: Mortality in Vermont granite workers and its association with silica exposure. Occupational and environmental medicine 2011, 68(5):312-318.
60. Wang Z, Dong D, Liang X, Qu G, Wu J, Xu X: Cancer mortality among silicotics in China's metallurgical industry. International journal of epidemiology 1996, 25(5):913-917.
61. Westberg H, Andersson L, Bryngelsson IL, Ngo Y, Ohlson CG: Cancer morbidity and quartz exposure in Swedish iron foundries. International archives of occupational and environmental health 2013, 86(5):499-507.
62. Westerholm P: Silicosis. Observations on a case register. Scandinavian journal of work, environment & health 1980, 6 Suppl 2:1-86.
63. Westerholm P, Ahlmark A, Maasing R, Segelberg I: Silicosis and risk of lung cancer or lung tuberculosis: a cohort study. Environmental research 1986, 41(1):339-350.
64. Wiebert P, Alderling M, Svartengren M, Gustavsson P, Plato N: 0225 Cancer, mortality and acute myocardial infarction in workers exposed to respirable crystalline silica dust at a Swedish porcelain factory. Occupational and environmental medicine 2014, 71(Suppl 1):A31.
65. Yu I, Tse LA, Chi CL, Tze WW, Cheuk MT, Alan CC: [A retrospective cohort study on mortality among silicotic workers in Hong Kong with emphasis on lung cancer]. Zhonghua lao dong wei sheng zhi ye bing za zhi = Zhonghua laodong weisheng zhiyebing zazhi = Chinese journal of industrial hygiene and occupational diseases 2008, 26(1):29-33.
66. Zambon P, Simonato L, Mastrangelo G, Winkelmann R, Saia B, Crepet M: Mortality of workers compensated for silicosis during the period 1959-1963 in the Veneto region of Italy. Scandinavian journal of work, environment & health 1987, 13(2):118-123.
67. Zhang X, Wang H, Zhu X, Liu Y, Wang L, Dai Q, Cai N, Wu T, Chen W: Cohort mortality study in three ceramic factories in Jingdezhen in China. Journal of Huazhong University of Science and Technology Medical sciences = Hua zhong ke ji da xue xue bao Yi xue Ying De wen ban = Huazhong keji daxue xuebao Yixue Yingdewen ban 2008, 28(4):386-390.
68. Bruske-Hohlfeld I, Mohner M, Pohlabeln H, et al. Occupational lung cancer risk for men in Germany: results from a pooled case-control study. Am J Epidemiol. 2000;151:384-395.
69. Cassidy A, t Mannetje A, van Tongeren M, Field JK, Zaridze D, Szeszenia-Dabrowska N, Rudnai P, Lissowska J, Fabianova E, Mates D et al: Occupational exposure to crystalline silica and risk of lung cancer: a multicenter case-control study in Europe. Epidemiology (Cambridge, Mass) 2007, 18(1):36-43.
70. Forastiere F, Lagorio S, Michelozzi P, Perucci CA, Axelson O: Mortality pattern of silicotic subjects in the Latium region, Italy. British journal of industrial medicine 1989, 46(12):877-880.
71. Fu H, Gu X, Jin X, Yu S, Wu K, Guidotti TL: Lung cancer among tin miners in southeast China: silica exposure, silicosis, and cigarette smoking. American journal of industrial medicine 1994, 26(3):373-381.
72. Kachuri L, Villeneuve PJ, Parent ME, Johnson KC, the Canadian Cancer Registries Epidemiology Group, Harris LA. Occupational exposure to crystalline silica and the risk of lung cancer in Canadian men. Int J Cancer. 2014;135:138-148.
73. Lagorio S, Forastiere F, Michelozzi P, Cavariani F, Perucci CA, Axelson O: A case-referent study on lung cancer mortality among ceramic workers. IARC scientific publications 1990(97):21-28.
74. Neuberger M, Westphal G, Bauer P: Long-term effect of occupational dust exposure. Sangyo igaku Japanese journal of industrial health 1988, 30(5):362-370.
75. Rodriguez V, Tardon A, Kogevinas M, Prieto CS, Cueto A, Garcia M, Menendez IA, Zaplana J: Lung cancer risk in iron and steel foundry workers: a nested case control study in Asturias, Spain. American journal of industrial medicine 2000, 38(6):644-650.
76. Samet JM, Pathak DR, Morgan MV, Coultas DB, James DS, Hunt WC: Silicosis and lung cancer risk in underground uranium miners. Health physics 1994, 66(4):450-453.
77. Schuler G, Walchi P, Ruttner JR, Delmore M, Taylor M, Schnieper R: [Incidence of lung cancer and age at death in silicosis deaths of the Swiss National Accident Insurance Fund, 1960-1978]. Sozial- und Praventivmedizin 1982, 27(5):218-219.
78. Tse LA, Yu IT, Qiu H, Au JS, Wang XR: Occupational risks and lung cancer burden for Chinese men: a population-based case-referent study. Cancer causes & control : CCC 2012, 23(1):121-131.
79. Tsuda T, Mino Y, Babazono A, Shigemi J, Otsu T,Yamamoto E et al. A case-control study of lung cancer in relation to silica exposure and silicosis in a rural area in Japan. Ann Epidemiol. 2002;12:288-294.
80. Ulm K, Waschulzik B, Ehnes H, Guldner K, Thomasson B, Schwebig A, Nuss H: Silica dust and lung cancer in the German stone, quarrying, and ceramics industries: results of a case-control study. Thorax 1999, 54(4):347-351.
81. Vida S, Pinto J, Parent ME, Lavoué J, Siemiatycki J. Occupational exposure to silica and lung cancer: pooled analysis of two case-control studies in Montreal Canada. Cancer Epidemiol Biomarkers Prev. 2010;19:1602-1611.
82. Watkins DK, Chiazze L, Jr., Fryar CD, Fayerweather W: A case control study of lung cancer and non-malignant respiratory disease among employees in asphalt roofing manufacturing and asphalt production. Journal of occupational and environmental medicine / American College of Occupational and Environmental Medicine 2002, 44(6):551-558.
83. Xu Z, Pan GW, Liu LM, Brown LM, Guan DX, Xiu Q, Sheng JH, Stone BJ, Dosemeci M, Fraumeni JF, Jr. et al: Cancer risks among iron and steel workers in Anshan, China, Part I: Proportional mortality ratio analysis. American journal of industrial medicine 1996, 30(1):1-6.
84. Kinlen LJ, Willows AN: Decline in the lung cancer hazard: a prospective study of the mortality of iron ore miners in Cumbria. British journal of industrial medicine 1988, 45(4):219-224.
85. Thomas TL: A preliminary investigation of mortality among workers in the pottery industry. International journal of epidemiology 1982, 11(2):175-180.
