# Supplementary material for: Occupational exposure to silica dust and risk of lung cancer: an updated meta-analysis of epidemiological studies
Source: BMC Public Health. 2016 Nov 4;16:1137. doi: 10.1186/s12889-016-3791-5 (PMC5095988; doi:10.1186/s12889-016-3791-5)
Supplement: Additional file 3 — Newcastle-Ottawa Scale (NOS) score for cohort and case-control studies. (DOC 141 kb) [file 12889_2016_3791_MOESM3_ESM.doc]

**Additional file 4 Newcastle-Ottawa Scale (NOS) score for cohort and case-control studies**

**Cohort studies**

|  | **Author, Year** | **Selection** | **Comparability** | **Assessment of outcome** | **Total Score** |
| --- | --- | --- | --- | --- | --- |
| 1 | Ahlman, 1991 | 3 | 2 | 1 | 6 |
| 2 | Ahn, 2010 | 0 | 2 | 2 | 4 |
| 3 | Amandus, 1995 | 1 | 2 | 1 | 4 |
| 4 | Andjelkovich, 1994 | 2 | 2 | 3 | 7 |
| 5 | Bergdahl, 2010 | 3 | 2 | 1 | 6 |
| 6 | Berry, 2004 | 0 | 2 | 2 | 4 |
| 7 | Brown, 2005 | 2 | 2 | 1 | 5 |
| 8 | Carta, 2001 | 2 | 2 | 3 | 7 |
| 9 | Chan, 2000 | 0 | 1 | 1 | 2 |
| 10 | Chen, 2012 | 3 | 2 | 3 | 8 |
| 11 | Chen, 2006 | 3 | 2 | 3 | 8 |
| 12 | Chen , 1992 | 2 | 2 | 3 | 7 |
| 13 | Chen , 1990 | 2 | 2 | 3 | 7 |
| 14 | Cherry, 2013 | 2 | 2 | 3 | 7 |
| 15 | Chia, 1991 | 1 | 2 | 0 | 3 |
| 16 | Chiyotani, 1990 | 0 | 2 | 1 | 3 |
| 17 | Cocco, 1994 | 2 | 2 | 2 | 6 |
| 18 | Costello, 1995 | 1 | 2 | 2 | 5 |
| 19 | Finkelstein, 1995 | 2 | 1 | 2 | 5 |
| 20 | Finkelstein, 2005 | 2 | 2 | 2 | 6 |
| 21 | Finkelstein, 1982 | 1 | 2 | 2 | 5 |
| 22 | Gallagher, 2005 | 2 | 2 | 2 | 6 |
| 23 | Giordano, 2011 | 2 | 2 | 2 | 6 |
| 24 | Goldsmith, 1995 | 0 | 2 | 2 | 4 |
| 25 | Graber, 2014 | 2 | 2 | 3 | 7 |
| 26 | Graham, 2004 | 0 | 2 | 1 | 3 |
| 27 | Guenel, 1989 | 1 | 2 | 1 | 4 |
| 28 | Hodgson, 1990 | 1 | 2 | 3 | 6 |
| 29 | Infard-Rivard, 1989 | 2 | 2 | 2 | 6 |
| 30 | Kauppinen, 2003 | 2 | 2 | 2 | 6 |
| 31 | Koh, 2011 | 1 | 2 | 2 | 5 |
| 32 | Koskela, 1994 | 2 | 2 | 2 | 6 |
| 33 | Kusiak, 1991 | 2 | 2 | 1 | 5 |
| 34 | Lawler, 1983 | 2 | 2 | 3 | 7 |
| 35 | Marinaccio, 2006 | 0 | 2 | 2 | 4 |
| 36 | Mehnert, 1990 | 1 | 2 | 1 | 4 |
| 37 | Meijers, 1996 | 1 | 2 | 2 | 5 |
| 38 | Merlo, 1991 | 0 | 2 | 3 | 5 |
| 39 | Merlo, 1995 | 2 | 2 | 1 | 5 |
| 40 | Miller, 2009 | 2 | 2 | 3 | 7 |
| 41 | Moulin, 1999 | 2 | 2 | 1 | 5 |
| 42 | Ng, 1990 | 1 | 2 | 2 | 5 |
| 43 | Olsen, 2012 | 2 | 2 | 3 | 7 |
| 44 | Partanen, 1994 | 1 | 2 | 2 | 5 |
| 45 | Peters, 2013 | 1 | 2 | 2 | 5 |
| 46 | Pham, 1983 | 2 | 2 | 1 | 5 |
| 47 | Preller, 2010 | 2 | 2 | 0 | 4 |
| 48 | Rafnsson, 1997 | 1 | 2 | 0 | 3 |
| 49 | Reid, 1996 | 2 | 2 | 2 | 6 |
| 50 | Scarselli, 2011 | 1 | 2 | 3 | 6 |
| 51 | Sherson, 1991 | 0 | 2 | 2 | 4 |
| 52 | Smailyte, 2004 | 2 | 2 | 2 | 6 |
| 53 | Steenland, 1995 | 3 | 2 | 3 | 8 |
| 54 | Steenland, 2001 | 2 | 2 | 3 | 7 |
| 55 | Thomas, 1990 | 1 | 2 | 1 | 4 |
| 56 | Tornling, 1991 | 0 | 2 | 1 | 3 |
| 57 | Tse , 2014 | 2 | 2 | 1 | 5 |
| 58 | Ulm, 2004 | 2 | 2 | 1 | 5 |
| 59 | Vacek, 2010 | 2 | 2 | 3 | 7 |
| 60 | Wang, 1996 | 1 | 2 | 2 | 5 |
| 61 | Westburg, 2013 | 3 | 2 | 2 | 7 |
| 62 | Westerholm, 1980 | 0 | 2 | 2 | 4 |
| 63 | Westerholm, 1986 | 0 | 2 | 1 | 3 |
| 64 | Wiebert, 2014 | 3 | 2 | 1 | 6 |
| 65 | Yu, 2008 | 2 | 2 | 1 | 5 |
| 66 | Zambon, 1987 | 0 | 2 | 1 | 3 |
| 67 | Zhang, 2008 | 3 | 2 | 2 | 7 |

**C**ase-control Studies

|  | **Author, Year** | **Selection** | **Comparability** | **Exposure ascertainment** | **Total Score** |
| --- | --- | --- | --- | --- | --- |
| 1 | Bruske-Hohfeld, 2000 | 3 | 2 | 2 | 7 |
| 2 | Cassidy, 2007 | 4 | 2 | 2 | 8 |
| 3 | Forastiere, 1989 | 1 | 2 | 2 | 5 |
| 4 | Fu, 1994 | 3 | 2 | 3 | 8 |
| 5 | Kachuri, 2013 | 3 | 2 | 2 | 7 |
| 6 | Lagorio, 1990 | 0 | 2 | 0 | 2 |
| 7 | Neuberger, 1988 | 4 | 2 | 2 | 8 |
| 8 | Rodriguez, 2000 | 3 | 2 | 1 | 6 |
| 9 | Samet, 1994 | 4 | 2 | 2 | 8 |
| 10 | Schuller, 1986 | 2 | 2 | 1 | 5 |
| 11 | Tse, 2012 | 3 | 2 | 2 | 7 |
| 12 | Tsuda, | 4 | 2 | 2 | 8 |
| 13 | Ulm, 1999 | 4 | 2 | 3 | 9 |
| 14 | Vida, 2010 | 3 | 2 | 2 | 7 |
| 15 | Watkins, 2002 | 3 | 2 | 1 | 6 |
| 16 | Xu, 1996 | 0 | 2 | 1 | 3 |
