# Supplementary material for: Occupational exposure to silica dust and risk of lung cancer: an updated meta-analysis of epidemiological studies
Source: BMC Public Health. 2016 Nov 4;16:1137. doi: 10.1186/s12889-016-3791-5 (PMC5095988; doi:10.1186/s12889-016-3791-5)
Supplement: Additional file 4: — Funnel plot for studies with standardized mortality ratio (SMR) as measure of association. (DOC 107 kb) [file 12889_2016_3791_MOESM4_ESM.doc]

**Additional file 5 Funnel plot for studies with standardized mortality ratio (SMR) as measure of association**

**
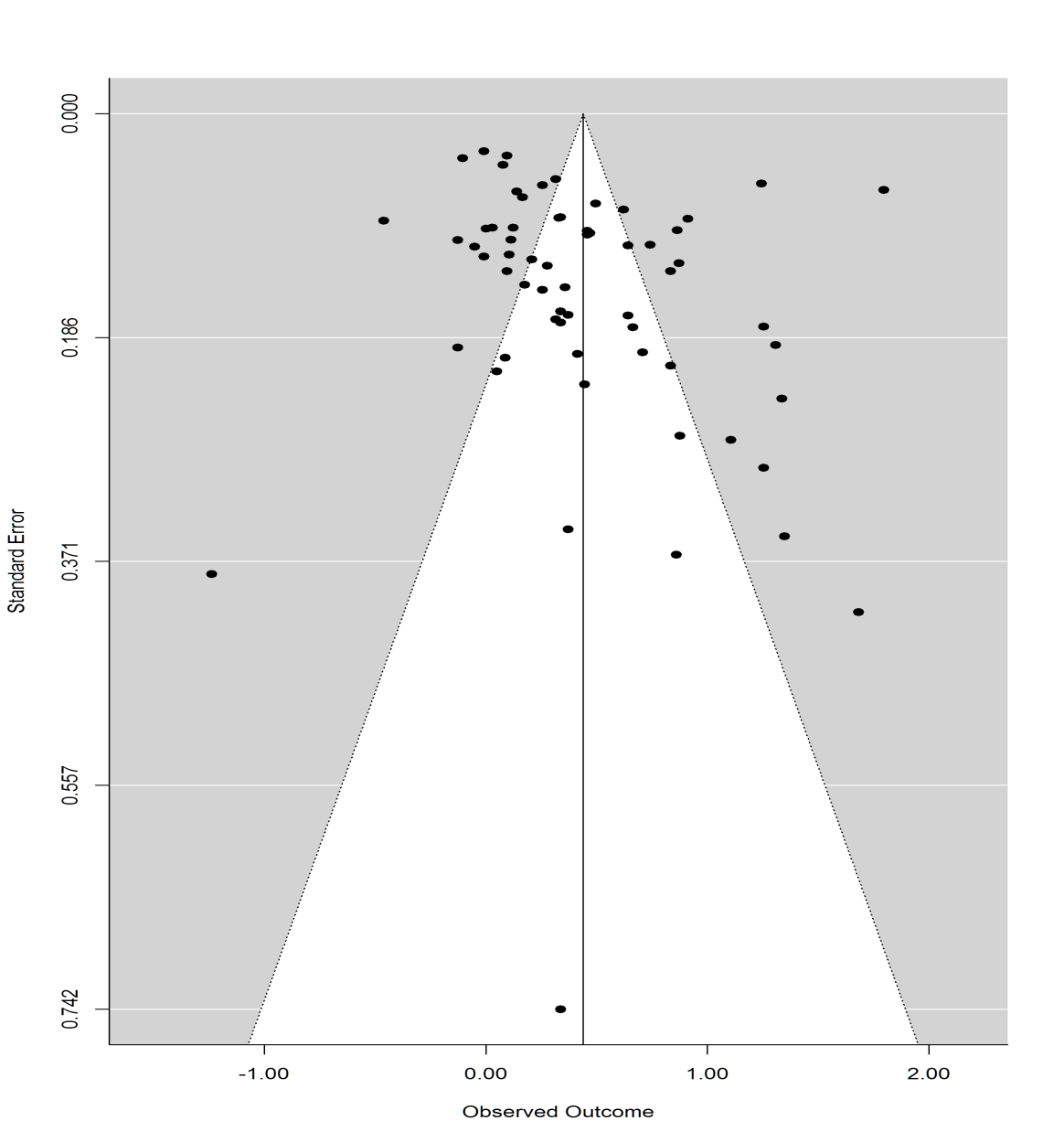
**
